# PLAIG: Protein–Ligand Binding Affinity Prediction Using a Novel Interaction-Based Graph Neural Network Framework

**DOI:** 10.1021/acsbiomedchemau.5c00053

**Published:** 2025-04-29

**Authors:** Madhav V. Samudrala, Somanath Dandibhotla, Arjun Kaneriya, Sivanesan Dakshanamurthy

**Affiliations:** † College of Arts and Sciences, 2358 The University of Virginia, Charlottesville, Virginia 22903, United States; ‡ College of Engineering and Computing, 3298 George Mason University, Fairfax, Virginia 22030, United States; § College of William and Mary, William and Mary, Williamsburg, Virginia 23185, United States; ∥ Department of Oncology, Lombardi Comprehensive Cancer Center, Georgetown University Medical Center, Washington, District of Columbia 20007, United States

**Keywords:** binding affinity, protein−ligand Interaction, graph neural network, drug discovery, ensemble learning, de novo prediction

## Abstract

Rapid prediction of protein–ligand binding affinity is important in the drug discovery process. The advent of machine learning methods has increased the speed of these predictions. Previous machine learning models based on structural, sequence, and interaction-based approaches have shown potential but often tend to memorize training data due to incomplete feature representations that lead to poor generalization on external complexes. To address this challenge, here, we developed PLAIG, a Graph Neural Network (GNN)-based machine learning framework for generalized binding affinity prediction. PLAIG represents binding complexes as graphs, integrating protein–ligand interactions and molecular topology to uniquely capture interaction and structural features. To reduce overfitting, we tested principal component analysis (PCA) and ensemble learning with a stacking regressor. During benchmarking, PLAIG achieved a PCC of 0.78 on 4852 complexes from the PDBbind v.2019 refined set and 0.82 on 285 complexes from the v.2016 core set, outperforming many existing models. External validation on the DUDE-Z data set demonstrated its ability to differentiate active ligands from decoys, achieving an average AUC of 0.69 and a maximum AUC of 0.89. To enrich de novo prediction capabilities for subsequent model versions, PLAIG was hybridized with sequence- and structure-based models. The hybrid models achieved an average PCC of 0.88 on well-known drug–target complexes, with the best reaching a PCC of 0.98. Future work will incorporate an explicit inclusion of a docking methodology into PLAIG’s pipeline and assess its performance on de novo ligands. PLAIG is freely available at https://plaig-demo.streamlit.app/.

## Graphical abstract

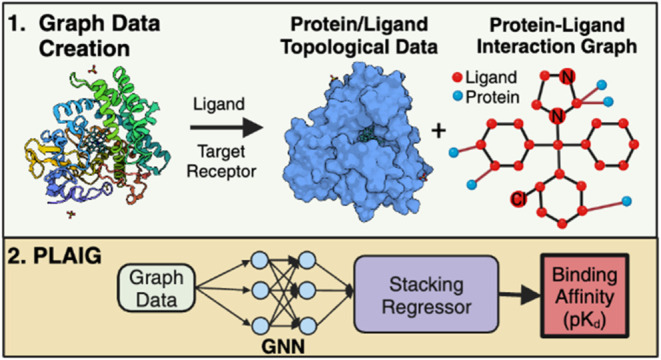

## 1 Introduction

Drug discovery and virtual screening rely on accurate protein–ligand binding affinity predictions to provide insights into the stability of interactions between potential drug candidates and their target proteins. Understanding these interactions is important for identifying compounds with the highest therapeutic potential while minimizing off-target effects.^1^ Accurate binding affinity predictions can significantly reduce the time and cost associated with experimental lab testing and clinical trials. Advances in computational techniques, including molecular docking and machine learning models, have improved the overall accuracy and efficiency of binding affinity predictions, making them an integral part of modern drug design studies.^2^

Deep learning frameworks, in particular, have emerged as a popular way to model the complex interactions between proteins and ligands. These methods use large data sets of known protein–ligand complexes to learn patterns about the features influencing binding affinity.^3^ One class of deep learning models, Convolutional Neural Networks (CNNs), have been used to capture spatial features of molecular interactions.^3,4^ For example, the CNN model *K*_DEEP_, by Jiménez et al., applies a 3D-CNN to a grid representation of the protein–ligand complex. *K*_DEEP_ achieves high accuracy on the PDBbind v.2016 core set.^5^ Likewise, we developed a hybrid neural network HNN-denovo that integrates a 3D-CNN and a fast forward neural network (FFNN).^6^ Recently, Graph Neural Networks (GNNs) have also been studied as a particularly promising approach due to their ability to naturally model the graph-like structure of a protein–ligand complex.^3^ Mqawass and Popov developed graphLambda, a deep learning model that inputs a graph representation of the ligand into GCN, GAT, and GIN blocks to predict binding affinity.^7^ GraphscoreDTA, created by Wang et al., uses a GNN combined with a bit-transportation mechanism and physics-based distance terms for this same task.^8^ These studies show the effectiveness of GNNs and CNNs as foundational models for protein–ligand binding affinity prediction.

Building on these base models, protein–ligand interaction-based methods further improve prediction accuracy. These methods focus on modeling the specific interactions at the protein–ligand interface, such as hydrogen bonds, hydrophobic interactions, and electrostatic interactions.^9–19^ For example, InteractionGraphNet (IGN) uses a deep graph representation to learn the intermolecular and intramolecular interactions of the protein–ligand complex.^20^ Zhang et al. developed SS-GNN, which uses a single undirected graph to model protein–ligand interaction features.^21^ By incorporating detailed interaction descriptors and using 3D representations of complexes, interaction-based models can capture the features that contribute to binding strength. This approach has led to significant improvements in prediction accuracy and has become a key focus in the development of rapid drug discovery tools.

Despite these recent advancements, deep learning models continue to make predictions with significant error when predicting the binding affinity specifically *d*e novo complexes.^18^ This poses a significant limitation for deep learning models, as their purpose is for rapid virtual screening of novel drugs to target proteins. There are two main reasons for this lack of generalization. First, models are trained on biased or homogeneous data sets, leading to inaccurate predictions since the model has never been exposed to any protein–ligand complex with similar properties during training.^22^ Second, incomplete feature representations interfere with learning.^3^ While data set bias is an important limitation to deep learning models, this study focuses on the lack of proper feature representation during training. Many interaction and structural-based models often use only atomic-level structural information for prediction. However, molecular binding requires accurate conformations. The global conformational data of the protein and ligand must be considered along with local features. The limited number of global features leads to increased overfitting during the training process, where the models tend to memorize the training data too well and cannot learn from its feature representations.^3^ This overfitting often leads to poor results when the model is exposed to novel data.^23^ These drawbacks emphasize the need for models that accurately predict de novo protein–ligand complexes by using both local protein–ligand interactions and global conformational information in the form of molecular topological data.

In this study, we addressed this limitation by developing a novel interaction-based GNN framework hybridized with XGBoost and Random Forest for protein–ligand affinity prediction. This framework, named PLAIG, converts the 3D coordinates of protein–ligand complexes into a series of graph-structured data. The graph-structured data objects have nodes composed of atoms and edges composed of chemical interactions between the different protein and ligand atoms. In addition, the graph holds topological and charge information on the individual molecules as graph-level data. The novelty of our approach is that both our hybridized GNN framework and the graph representation of the protein–ligand complex include interaction and molecular shape features stored in a unique way. In addition, we combined our framework with separate structure-based and sequence-based models to capture a larger number of features needed to predict binding affinity. Our train-test and external validation results indicate that PLAIG outperforms existing models and has the potential to make robust predictions for de novo protein–ligand complexes.

## 2 Materials and Methods

### 2.1 Data Sets

For the initial training, testing, and external validation, we collected protein–ligand binding complexes from the “refined set” and “general set” of the PDBbind v.2020 database. The refined data set consists of 5320 experimentally validated binding affinity values for protein–ligand complexes described in the RCSB Protein Data Bank, while the general data set consists of 14,127 protein–ligand complexes. The binding affinity values in the refined set are annotated as either a negative base-10 log transformation of the *K*_d_ dissociation or the *K*_i_ inhibition constant (p*K*_d_ and p*K*_i_), while the general set includes complexes annotated by an IC_50_ value as well. In our models, we predict the ligand binding affinity as the p*K*_d_ or p*K*_i_ (−log­(*K*_d_/*K*_i_)). Thus, before using the general set, we filtered out the complexes with IC_50_ values and kept only those with p*K*_d_ or p*K*_i_ constants. The protein–ligand complexes are split up as four different files to represent 3D coordinates of either the protein or ligand:. mol2 and .sdf files for the ligand, a .pdb file for the entire protein, and a .pdb file for atoms in just the binding pocket. Only the .mol2 ligand file and the .pdb protein binding pocket file were used for feature extraction and model training/validation. To identify the optimal model using subsets from the PDBbind v.2020 data set, we developed and evaluated three distinct types of models. The first model was trained on 5200 complexes from the refined subset following the removal of any sanitization and conversion errors. The second model used the general set, while the third model was trained on a randomized combination of the refined and general subsets deemed the “combined set”. The exact training and testing data sets used for PLAIG are available in the folder named “Refined_General_Files” at the GitHub repository: https://github.com/sivaGU/PLAIG.

The data set for our secondary external validation was sourced from the newly compiled DUDE-Z database. This database includes 3D-coordinate files for 43 receptors, as well as thousands of active ligands and artificial decoys with chemical properties similar to the actives.^24^ We selected the DUDE-Z database because it provides predocked ligands and decoys with the receptors, which is required for our interaction-based model. This model requires docked complexes to calculate the chemical interactions and energies between the molecules. The DUDE-Z database offers three distinct sets of decoys for each receptor: “regular” decoys from the DUD-E database, “goldilocks” decoys representing molecules with average properties, and “extrema” decoys designed to exhibit extreme chemical interactions. The receptors were provided in .pdb format, while the ligands and decoys were in .mol2 format. Similar to our training and test sets, we used the prepare_receptor4.py and prepare_ligand4.py from MGLTools to convert these files into .pdbqt and .pdb formats for use in our model.^25^

### 2.2 Creating Protein Pocket Substructures Using Distance-Based Pocket Extraction

Although the PDBbind data set provides. pdb files for the protein binding pockets, external validation sets such as the DUDE-Z data set do not include protein pocket structures. In addition, most users may not have access to the .pdb format of the protein’s binding pocket structure, which is required for binding affinity prediction with PLAIG. To address this, we extracted the protein binding pocket using the full protein structure and the docked ligand file. With the Python package Biopython, we generated a custom PDB file representing a substructure of the protein that includes only the residues within 10 Å of the hydrogenated ligand in its docked conformation. The protein substructures, representing the 3D coordinates of the binding pocket, were saved and used for calculating interaction features and topological structural features. By focusing solely on the binding site and excluding residues unrelated to binding affinity, this approach significantly reduced the computational time required for graph creation. Figure S1 illustrates an example of a protein substructure extracted from a full protein file, representing the binding pocket. This figure can also be viewed in the Graph Representation tab at https://plaig-demo.streamlit.app/Graph_Representation.

### 2.3 Obtaining Protein–Ligand Interaction Features Using BINANA

We used the protein–ligand interactions calculated by the BINANA molecular descriptor tool as input for our GNN model.^26^ The ligand .mol2 and the protein binding pocket. pdb files were converted to .pdbqt format using the prepare_ligand4.py and prepare_receptor4.py scripts from AutoDock’s MGLTools package.^25^ BINANA then computed the number and locations of various chemical interactions between the protein and ligand atoms, including electrostatic energies, hydrogen bonds, halogen bonds, hydrophobic contacts, metal contacts, π–π stacking, T-stacking, salt bridges, and cation–π interactions. BINANA calculates these interactions within a user-defined distance. After testing cutoff distances of 2, 3, 4, and 5 Å, we determined that a cutoff distance of 3 Å was optimal for this study. A detailed description of how we incorporated BINANA into our Python code is provided in the webpage app located at https://plaig-demo.streamlit.app/. A complete list of BINANA descriptors and how they are stored within our protein–ligand graph representations can be found in Table S1.

### 2.4 Creating Graph Representations of the Protein–Ligand Complex

To input data into the GNN framework, we created graph representations of the protein binding pocket-ligand complexes using the Networkx Python library. The .pdbqt files used for protein–ligand interaction analysis were converted into .pdb files using OpenBabel. RDKit was then used to read in the pocket and ligand data from the .pdb files. For the graph representation, all ligand atoms were represented as nodes, while only a select few protein atoms within the specified distance cutoff of 3 Å from any ligand atom were included as nodes. This selection ensured that the majority of included protein atoms had a significant chemical interaction with the ligand, maximizing the information extracted while minimizing computational time during training.

After modeling the protein–ligand complex, atomic structure and interaction features were added to the graph as node and edge attributes. Each node (atom) was assigned an array consisting of ligand or protein structural features, along with its 3D-coordinates. The edge features were included as an array with values of 0 or 1, indicating the presence of a particular chemical interaction described in Section 2.2. Additionally, the edges included the bond type and the distance in angstroms between two atoms. Due to the cutoff distance of 3 Å, the resultant protein–ligand graphs are inherently sparse. In each graph, edges exist between ligand atoms to form the ligand’s molecular structure, as well as between outer ligand atoms and nearby protein pocket atoms. However, edges do not connect all node pairs, as a fully connected graph would significantly reduce computational efficiency. Instead, BINANA calculates edge features only for protein pocket atoms within 3 Å of any ligand atom, ensuring that only relevant interactions are captured. Rather than explicitly introducing additional edges, we assign multiple edge features to each existing edge. PLAIG uses these edge features for binding affinity predictions. Tables 1 and 2 list the node and edge features included in each graph representation of a protein binding pocket-ligand complex.

**Table 1:** List of Node Features Stored in a Graph Representation of a Protein–Ligand Complex

feature	representation in node
atomic number	integer
residue or ligand name	one-hot encoded
hybridization number	one-hot encoded
degree	integer
aromaticity	0 if not, 1 if yes
number of hydrogens	integer
atomic mass	float
formal charge	integer
gasteiger charge	float
*XYZ* coordinates	float

**Table 2:** List of Edge Features Stored in a Graph Representation of a Protein–Ligand Complex

feature	representation in edge
electrostatic energy	float
halogen bond	0 if not, 1 if yes
hydrogen bond	0 if not, 1 if yes
hydrophobic contact	0 if not, 1 if yes
metal contact	0 if not, 1 if yes
π–π stacking	0 if not, 1 if yes
T-stacking	0 if not, 1 if yes
salt bridge	0 if not, 1 if yes
cation–π	0 if not, 1 if yes
bond type	0 if protein–ligand interaction, integer if ligand bond
distance (Å)	float

We performed *z*-score standardization on the electrostatic energies between atoms and the Gasteiger partial charges of each atom. Min–max normalization was applied to the distances between atoms and the masses of each atom, ensuring consistent ranges among all features. Additionally, categorical features such as hybridization and residue name were one-hot encoded into the nodes for streamlined input into the model. The node and edge feature arrays were cast as float-type PyTorch tensors before being added to the graph.

In addition to atom-level structural features, we included ligand and protein pocket structural features as global graph-level features. These features characterize the hydrophobicity, surface area, and topological properties of the protein and ligand. They are important for assessing how well a ligand fits into a receptor binding pocket. Like the atom-level features, the global graph features were standardized using z-scores and converted to float-type PyTorch tensors before being input into the model to prevent any single feature from skewing the results. In total, our model uses 40 node features, 11 edge features, 88 global ligand features, and 74 global pocket features. Table S1 lists all the node, edge, and graph-level features included in each graph representation. Figure S2 is an example visualization of a graph representing a protein–ligand complex. More information on creating graph representations of the protein–ligand complex can be found at https://plaig-demo.streamlit.app/Graph_Representation.

### 2.5 Principal Component Analysis

After generating graph representations of the protein–ligand complex, we optimized the number of topological structural features included in the graph-level data using Principal Component Analysis (PCA). The objective of PCA was to transform the original set of topological features into a smaller set of principal components. This reduces the complexity of the data while retaining as much relevant information as possible about the binding affinity. We attempted PCA to minimize overfitting and improve the robustness of the model. Initially, we normalized the topological features using z-scores to make sure the features had consistent ranges. We then applied PCA to both the ligand and pocket topological features using the Sklearn library. Next, we used Matplotlib to visualize the explained variance of the data as the number of features increased. This plot showed how much of the total information about binding affinity was retained with each additional feature. By plotting the cumulative explained variance against the number of features, we identified the “elbow point”—the point where the information gain leveled off. This allowed us to reduce the dimensionality of the data by keeping only the number features up to the elbow point. The thought was that if we kept more features past the elbow point, it would have only minimally helped the model predict the binding affinity of a certain complex. Determining the elbow point with PCA allowed us to remove potentially redundant features to maximize efficiency.

### 2.6 Optimization of GNN Models

Once feature engineering with PCA was complete, we moved to designing PLAIG’s GNN framework. We performed hyperparameter optimization via the Tree-Structured Parzen Estimator (TPE) within the Hyperopt python package. Instead of brute force testing the given search space of parameters, TPE works by choosing the next set of hyperparameters to test based on conditions that are similar to successful hyperparameters in previous iterations. Table 3 shows the search space of parameters tested using Hyperopt.

**Table 3:** Search Space of Hyperparameters Tested during GNN Optimization with Hyperopt

parameter	search space
hidden channels	64, 128, 256
GNN layers	2, 3, 4, 5
dropout rate	0.2, 0.3, 0.4, 0.5
learning rate	0.001, 0.005, 0.01, 0.05
batch size	32, 64, 128, 256
epochs	50, 75, 100

To identify the optimal set of hyperparameters, Hyperopt minimizes an objective function. Our primary metric for evaluating model performance was the Pearson Correlation Coefficient (PCC). During the hyperparameter tuning process, we used 5-fold cross-validation to assess different sets of hyperparameters. For each set, we calculated the average PCC across all folds. Since Hyperopt operates by minimizing the objective function and higher PCC values indicate a higher correlation between the predicted and actual values, we provided Hyperopt with the negative PCC values to guide its search for the best set of hyperparameters. We configured Hyperopt to evaluate a maximum of 20 hyperparameter combinations. Therefore, out of all possible configurations, Hyperopt explored 20 distinct sets of hyperparameters before selecting the optimal set which is described in Section 2.7. We also used Hyperopt to optimize hyperparameters for the hybridized versions of PLAIG with external models, discussed in Section 2.8. Specifically, we tuned two key hyperparameters: the number of fully connected layers for the sequence and structure features, as well as the number of hidden channels in these fully connected layers. The search space for the hidden channels was 64, 128, and 256, while the number of fully connected layers were 1, 2, 3, 4, and 5.

### 2.7 Graph Neural Network Models

Our GNN-based deep learning model was developed using the PyTorch-Geometric library, which handles graph-structured data efficiently. The graphs were converted into PyTorch-Geometric Data objects, allowing the GNN to distinguish between various types of features. In addition, the graphs were processed in batches of 32, as smaller batch sizes improve generalization. The training stage of the GNN framework begins with an input convolutional layer (GeneralConv) that processes 40 node channels and 11 edge channels, corresponding to the different features. These features are mapped to 256 hidden channels and processed through three additional convolutional layers, each also mapped to 256 hidden channels. The layers accept PyTorch float tensors containing atom structural features and BINANA interaction descriptors of the pocket-ligand complex embedded in graphs. Each convolutional layer applies the ReLU activation function to introduce nonlinearity.

After passing the node and edge features through a total of four convolutional layers, the embeddings are aggregated to the graph level using global mean pooling. The global ligand and protein pocket structural features are each processed through their own fully connected layer with 256 hidden channels in parallel. The outputs from these layers, along with those from the convolutional layers, are concatenated and passed through a fully connected layer of size 256 to generate the final embeddings. To avoid overfitting, we processed these final embeddings through a dropout layer with a rate of 0.2. Finally, the embeddings were put through a final Linear layer to obtain binding affinity predictions for each protein–ligand complex. After obtaining binding affinity predictions, the GNN framework computes the mean absolute error (MAE) of the predicted and experimental values as the loss and employs the Adagrad optimizer for gradient-based weight updates. The model was trained with a learning rate of 0.001 for 50 epochs.

Another step in the training process was used to train the stacking regressor that combines the XGBoost and RF regressors. Hybridizing the GNN with these ensemble models enhances overall performance by reducing overfitting and assessing feature importance. To train the stacking regressor, we put the protein–ligand graphs through our already trained GNN framework to receive outputs. However, instead of generating singular value affinity predictions, we output the embeddings that are created just before the final Linear layer. These embeddings and the experimental binding affinity values are then used to train the stacking regressor. Figure 1 shows the training framework of PLAIG.

**Figure 1:**
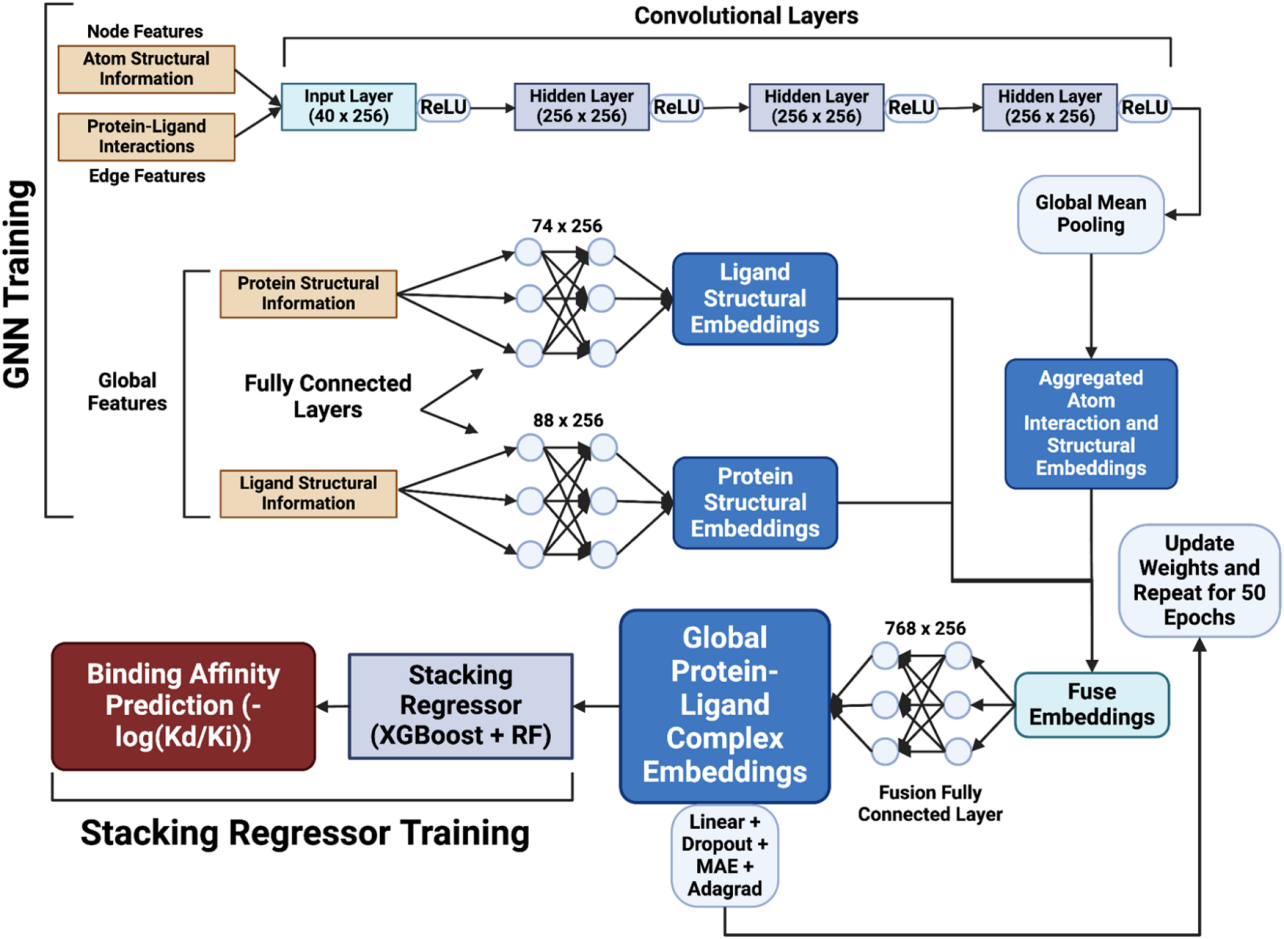
Training framework for PLAIG. The framework involved two parallel pathways: one for node and edge features with atomic structural and interaction data, and another for graph-level features with protein and ligand topological structure data (74 and 88 features, respectively). Node and edge features were passed through four convolutional layers and were then aggregated to the graph level using global mean pooling. The global features were processed through fully connected layers to generate embeddings, which were fused with the aggregated embeddings from the first pathway. After a final fully connected layer, a linear layer and dropout generated binding affinity predictions, with MAE as the loss function and Adagrad as the optimizer. The GNN was trained for 50 epochs. Subsequently, a stacking regressor was trained using GNN embeddings (after 50 epochs of training) as input features and experimental binding affinities as targets. PLAIG’s final binding affinity predictions in −log­(*K*_d_/*K*_i_) units are derived from uniquely structured graph data processed by the hybridized GNN and stacking regressor.

### 2.8 Integration of External Models for Improved Affinity Predictions

The primary goal of protein–ligand binding affinity predictions is to leverage in-silico methods for rapid virtual screening and potential drug discovery of de novo complexes. To increase the accuracy of these predictions, we developed a small subset of models that were hybridized with PLAIG. The key distinction between these hybrid models and PLAIG lies in their focus on different types of features. The two additional models we integrated with are specialized in extracting sequence-based and structural features, as opposed to interaction-based features. The sequence-based model employs a GNN to derive features from the amino acid sequence of the protein pocket and the SMILES format of the ligand. In contrast, the structural model constructs two separate graphs, one for the protein and one for the ligand, concatenates their respective structural features, and then processes them through a GNN.

We hybridized PLAIG with the sequence-based model using three distinct approaches. First, we extracted sequence features from the GNN of the sequence model, passed them through a fully connected layer, and concatenated these features with PLAIG’s GNN embeddings. The resulting tensor was fed into a final fully connected layer, followed by a hybrid of Random Forest and XGBoost for a singular affinity prediction. The second method was based on a stacking procedure. We concatenated the output features from the sequence model with PLAIG’s embeddings and applied a stacking regressor combining Random Forest and XGBoost to make predictions. In the third approach, instead of concatenating embeddings and features, we performed stacking directly on the singular predictions from both PLAIG and the sequence-based model.

For the structure-based model, we used a similar method as the first approach with the sequence model. To avoid redundancy between the node and edge features from the structure model’s protein and ligand graphs, we extracted solely the global structural features from the structure-based model, passed them through a fully connected layer, and concatenated them with PLAIG’s embeddings. This combined representation was processed through another fully connected layer to generate the final affinity embeddings, which were hybridized with Random Forest and XGBoost for singular predictions. A separate stacking procedure was not implemented for the structure-based model due to its weaker standalone predictions. More importantly, the structural model provides an extremely rich set of molecular fingerprints and topological features, which we believed could potentially improve upon the standalone predictions of PLAIG. These hybridization strategies were designed to use the strengths of each model and improve the accuracy of binding affinity predictions for de novo complexes in future studies. Figure 2 displays a schematic that delineates the approaches taken to hybridize PLAIG with the sequence- and structure-based models.

**Figure 2:**
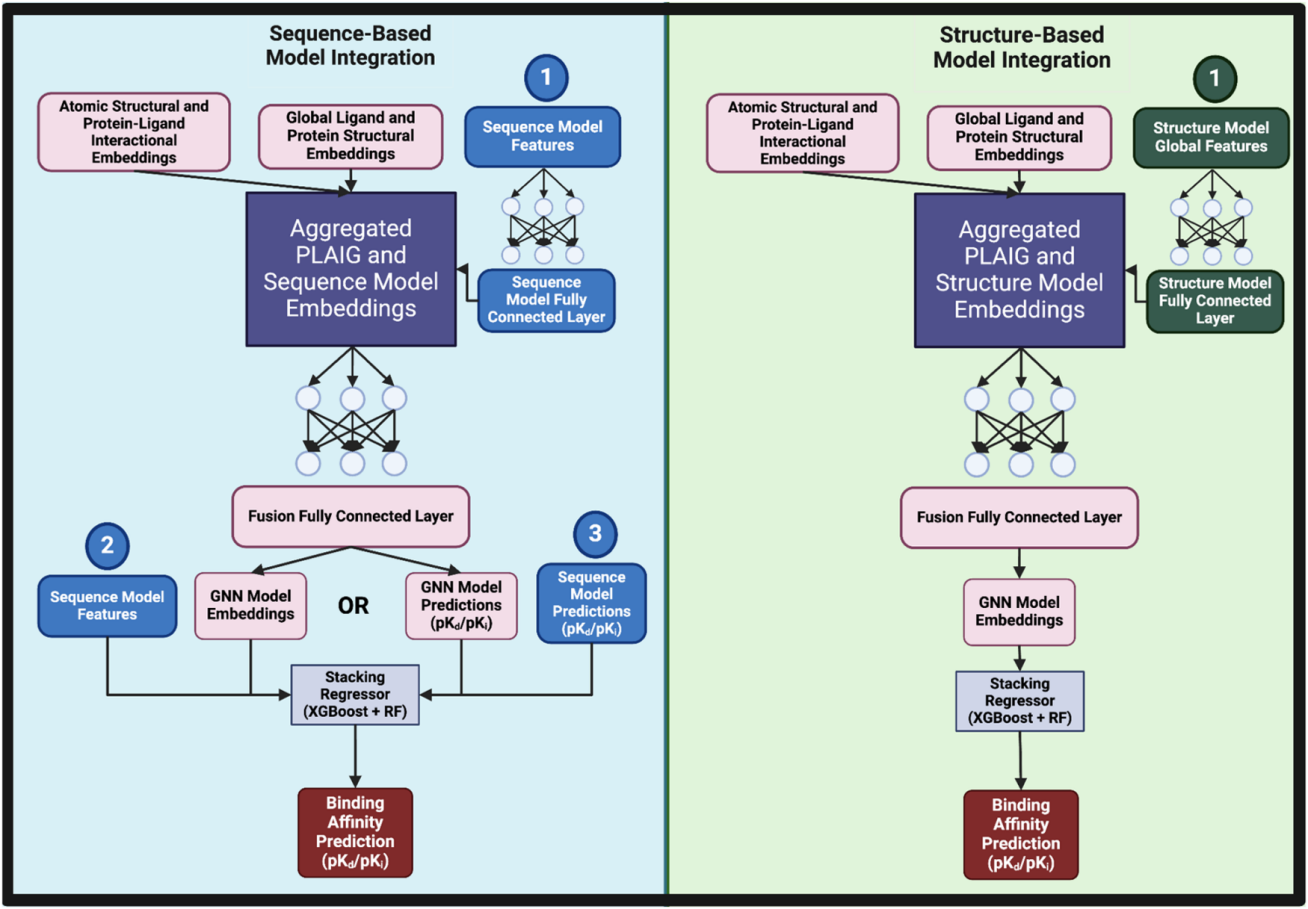
Schematic diagram differentiating between the methods used to hybridize PLAIG with the sequence- and structural-based models. For sequence-based hybridization, we explored three approaches. In Path 1, atomic structural, protein–ligand interaction, global structural, and sequence model features were combined into a single tensor, passed through a fully connected layer, and output as GNN embeddings. These embeddings were then processed by a stacking regressor for binding affinity prediction. In Path 2, sequence model features were excluded from the GNN but concatenated with the GNN’s output embeddings before being fed into the stacking regressor. In Path 3, instead of embeddings, binding affinity predictions from both the GNN and sequence model were concatenated and input into the stacking regressor. For structure-based hybridization, we followed an approach similar to Path 1 in the sequence-based model, where global embeddings from the structure-based model were aggregated with PLAIG’s embeddings before continuing through the model’s framework.

### 2.9 Outlier Removal

To improve model performance and robustness, we removed outliers from the training data set by comparing the model’s predicted values with the actual values provided by PDBbind. Specifically, we excluded protein–ligand complexes whose prediction errors exceeded 3 standard deviations (*z*-scores). Outliers can lead to biased results and reduced model accuracy. By removing these outliers, we ensured a higher-quality data set, ultimately contributing to better model performance and greater reliability.

## 3 Results and Discussion

### 3.1 PLAIG Model Development Workflow

We developed PLAIG, an interaction-based Graph Neural Network (GNN) hybridized with XGBoost and Random Forest, to predict protein–ligand binding affinity. The workflow for this model, shown in Figure 3, consists of two phases: graph representation and model evaluation. In the graph representation phase, we prepared three data sets from PDBbind v.2020: the refined data set, the general data set, and a combined data set. The combined data set excludes ∼5000 random complexes for faster computational time during training. Using the BINANA Python library, we computed chemical interactions between the protein binding pocket and the ligand. These interactions, such as hydrogen bonds and π–π stacking, directly influence binding stability and free energy. As binding affinity can be calculated from the binding free energy, the presence and location of protein–ligand interactions are key information for PLAIG.^27,28^ However, interaction features by themselves cannot characterize the binding of a protein to its ligand. The structural conformation of the binding pocket is important to determine molecular fit.^29^ Therefore, accurate calculation of binding affinity requires considering both the chemical interactions and the structural topology of the protein–ligand complex.

**Figure 3:**
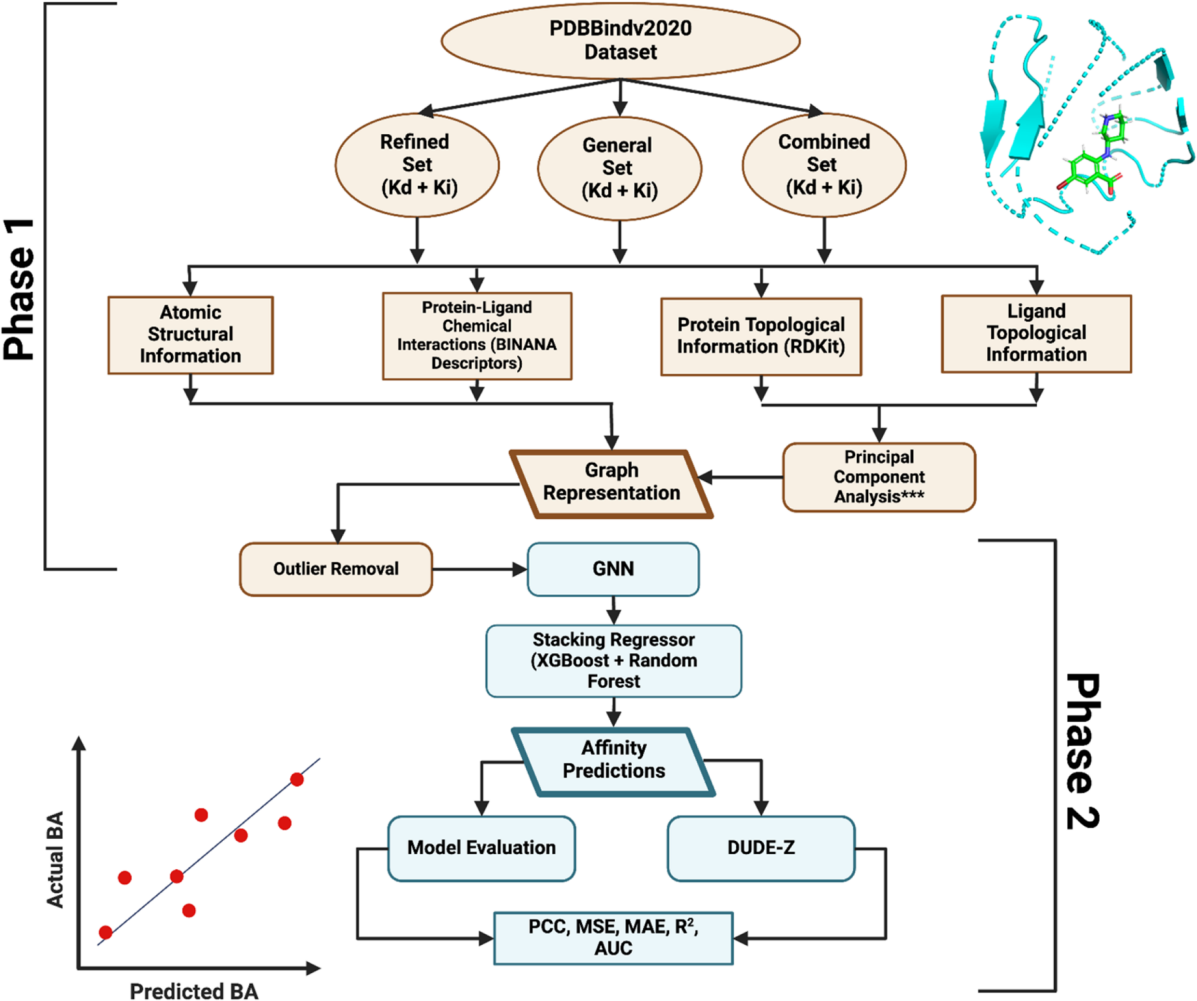
Overall workflow design of this study. This workflow outlines the process of creating diverse graph representations using the PDBbind v.2020 data set, followed by the development of GNN deep learning models, and concluding with external validation using hold-out data sets from PDBbind v.2020 and DUDE-Z. The protein pocket-ligand complex shown corresponds to RCSB code 3IP5. The asterisks in the Principal Component Analysis (PCA) block indicate that separate PLAIG models were developed using PCA. However, training and testing metrics indicated that PLAIG with PCA performs badly on external data sets. Therefore, PCA is not included in the final model framework.

To capture these structural features, we used RDKit to extract atomic properties and topological descriptors of the ligand and protein binding pocket. This combination of features embedded into the graph representation allows the model to capture both the detailed interactions and the overall molecular fit of the complex. To streamline features and reduce redundancy, we performed principal component analysis (PCA). This analysis allowed us to identify and eliminate potentially redundant topological features. Redundant features can complicate the model and increase the risk of overfitting. Since the goal of this study is to create a model that performs well on de novo complexes without overfitting, we compared the performance of models with and without feature optimization using PCA to assess the impact of removing these features. It is important to note that PCA was used during the model development workflow but is not part of PLAIG’s final model framework, as shown in Figure 1. Models with and without PCA were tested, but those incorporating PCA performed significantly worse during external validation. As a result, PCA was excluded from the final model framework.

In the model evaluation phase, the graph-structured data was input into PLAIG for training and validation. PLAIG uses convolutional layers to analyze spatial patterns in the chemical interactions and fully connected layers to process global structural features. The model synthesizes these features into global embeddings, which are passed to a stacking regressor combining Random Forest and XGBoost. Again, since one of the main goals of this study is to create a model capable of making accurate de novo predictions, we used RF and XGBoost to improve the robustness, generalization, and reduce overfitting of the GNN model. The final steps in our experimental design process are the validation of our three models using external data sets, such as subsets from the PDBbind v.2020 data set that were not present during training and the DUDE-Z data set. The DUDE-Z data set is used to test a model’s ability to discern between active and decoy ligands.^24^ The following results of this study demonstrate the predictive capabilities of PLAIG. In addition, these results provide evidence that our novel graph-structured data, which models the protein–ligand binding interface, contains valuable information for binding affinity prediction studies.

### 3.2 Principal Component Analysis (PCA)

Before evaluating PLAIG, we applied Principal Component Analysis (PCA) to reduce dimensionality of the input features and create a more robust model. We used PCA for each subset of the PDBbind v.2020 data set that we trained and tested on (refined, general, and combined). In order to determine how many features to reduce down to, we generated six graphs to illustrate the cumulative explained variance as a function of the number of features. We created separate graphs for ligand features and protein features across the three subsets of the PDBbind v.2020 data set. These graphs were analyzed to identify the “elbow point,” as detailed in Section 2.5, which indicates the optimal number of features for effective dimensionality reduction. Figure 4 shows two of these graphs created from the refined set. The other four graphs for the general and combined sets are presented in Figures S3 and S4. After examining the graphs, we determined that the optimal reduction for the 88 topological ligand features was to 22 features, while the 74 topological protein features were best reduced to 13 features.

**Figure 4:**
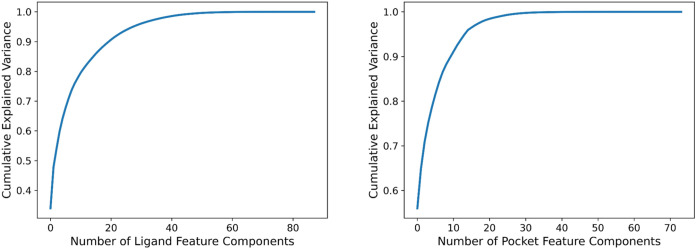
Cumulative explained variance plots which indicate the percentage of cumulative variance explained by the number of topological feature components for the ligand and protein. The figure on the left is the plot for the ligand features, while the figure on the right is for the protein pocket features. The “elbow” point of each graph shows the ideal number of principal components or features needed to capture the necessary information.

### 3.3 Model Evaluations Based on the PDBBind v.2020 Data Set

To evaluate the performance of each model after training, we used 10-fold cross-validation with an 80:20 training-to-testing split. We assessed each version of PLAIG using several metrics: Pearson Correlation Coefficient (PCC), Mean Squared Error (MSE), Mean Absolute Error (MAE), *R* ^2^, and Area Under the Curve (AUC). PCC measures the linear correlation between predicted and actual values, with values closer to 1 indicating stronger correlation and more accurate predictions. MSE represents the average squared difference between predicted and actual values, with lower values reflecting better model performance. MAE calculates the average magnitude of errors between predicted and actual values, without considering their direction, and lower MAE values indicate better model accuracy. *R* ^2^ indicates the proportion of variance in the actual values explained by the model, with higher values closer to 1 suggesting that the model captures more of the data’s variance. AUC measures the model’s ability to distinguish between two classes, reflecting how well the model discriminates between low and high binding affinities. AUC values closer to 1 indicate better discrimination, with low binding affinity values below the median and high binding affinity values above the median. We also recorded the training runtimes for each fold, as longer runtimes generally indicate more complicated models. All PLAIG training and testing simulations were performed on a system equipped with an Apple M1Max CPU and 64 GB of RAM.

#### 3.3.1 Model Evaluation Based on the Refined Set

The first pair of models were trained and tested on the refined set from the PDBbind v.2020 data set. The model without PCA contained 88 topological ligand features and 74 topological protein features, while the model with PCA applied had reduced dimensionalities of 22 ligand features and 13 protein features. Figure 5 shows the evaluation metrics of each model over 10 splits of training and testing on the refined set (10-fold cross-validation), as well as the runtime for each fold using the system performance metrics detailed in Section 3.3.

**Figure 5:**
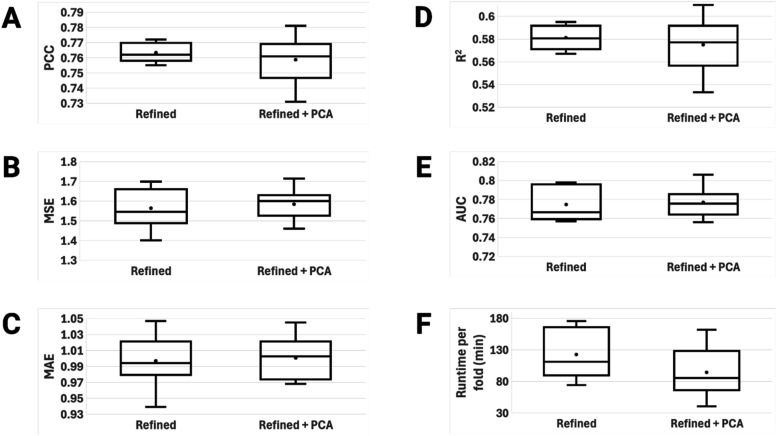
PLAIG’s performance metrics for the refined set comparing the model before and after principal component analysis was implemented. (A) PCC, (B) MSE, (C) MAE, (D) *R* ^2^, (E) AUC, (F) Runtime per fold (min).

Across the 10-fold cross-validation, PLAIG had average PCCs of 0.763 and 0.759, average MSEs of 1.564 and 1.584, average MAEs of 0.997 and 1.001, average *R* ^2^s of 0.581 and 0.575, and average AUCs of 0.774 and 0.777 for the pre-PCA and post-PCA models, respectively. Collectively, these metrics confirm that the models consistently delivered strong performance in predicting the binding affinity of protein–ligand complexes in the refined set. Comparing the two models against each other, we can see that the performance metrics are virtually the same. This is because PCA tries to retain as much information in the input data as possible while attempting to reduce overfitting. Any significant changes in accuracy or performance will show in external validation steps rather than training and testing. However, one difference between the two models that we can observe is the runtime for the 10-fold cross validation. As we can see in Figure 5, the F boxplot shows that the average runtime per fold of the model with PCA applied is lower than the runtime per fold of the model without PCA, 94.3 min versus 122.6 min. This is because PCA reduces the complexity of the overall model by reducing the number of features, thus making the model faster.

#### 3.3.2 Model Evaluation Based on the General Set

The next two models were trained and tested on the general set from the PDBbind v.2020 data set. Again, like the two models tested above, the model without PCA learned on graph data with 88 topological ligand features and 74 topological protein features, while the model with PCA applied had dimensionalities of 22 ligand features and 13 protein features. Figure 6 shows the performance metrics for these two models in side-by-side boxplots after 10-fold cross validation was conducted, along with boxplots of the runtime per fold in minutes.

**Figure 6:**
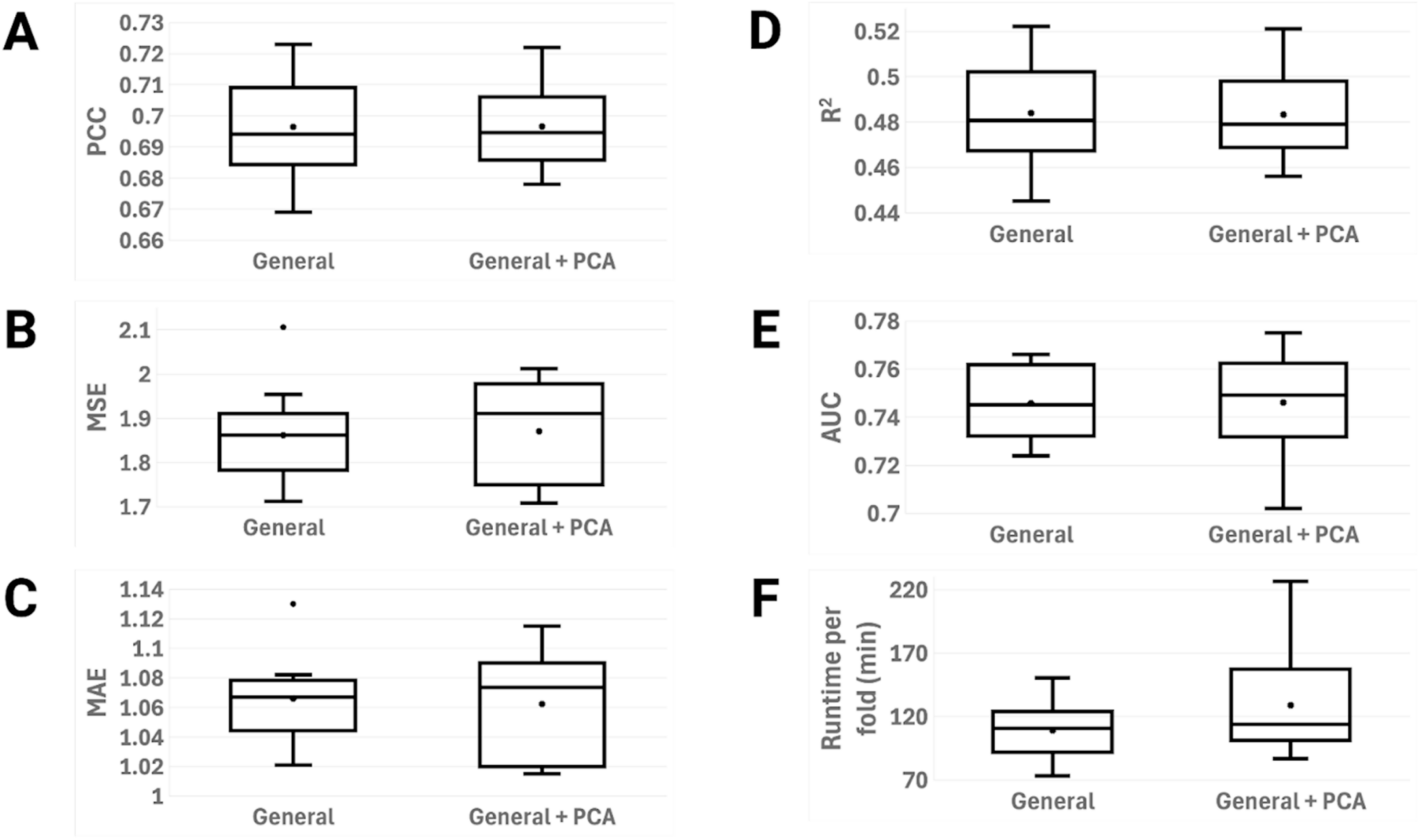
PLAIG’s performance metrics for the general set comparing the model before and after principal component analysis was implemented. (A) PCC, (B) MSE, (C) MAE, (D) *R* ^2^, (E) AUC, (F) Runtime per fold (min).

After the simulations were completed on the general set, PLAIG had average PCCs of 0.696 and 0.697, average MSEs of 1.862 and 1.870, average MAEs of 1.066 and 1.062, average *R* ^2^s of 0.484 and 0.483, and average AUCs of 0.745 and 0.746 for the pre-PCA and post-PCA models, respectively. Similar to the models trained on the refined set, there is no significant difference between the performance of these two models trained on the general set. The average runtimes for the pre- and post-PCA models were 108.9 and 128.8 min, respectively. This is a change from what we observed for the refined set models, as the model with PCA has a longer runtime. Although this is the case, looking at the General + PCA boxplot in section F of Figure 6, we can see that the runtimes for the 10 folds are right skewed and have a large range. Based on this observation, we cannot say that the difference in runtime is significant, and we cannot generalize that the model with PCA is more or less complex than without PCA.

More importantly than comparing the two general set models against each other is the comparison of PLAIG trained on the general set with PLAIG trained on the refined set. Except for the runtime, all of the performance metrics are significantly higher when PLAIG is trained and tested on the refined set rather than the general set. A possible explanation for this is that the general set is a much more diverse and complex data set than the refined set. It contains protein–ligand complexes with binding affinity values ranging from mM to fM, while the refined set has values from mM to nM. In addition, the general set contains approximately 14,000 complexes filtered down to 6000 complexes versus the refined set with merely 5000 complexes before filtering. When the diversity of the data increases, this introduces new complexities or variations that the model struggles to capture. In the case of the general and refined sets, PLAIG is able to predict the affinity of complexes from the refined set with greater ease since it does not need to accurately model as many complex biochemical relationships and generalize over a larger range of binding affinity values. Another explanation is that the resolution or quality of. pdb files is sharper in the refined set than the general set. More accurate protein–ligand crystal structures lead to more accurate feature representations.^30^ This is particularly important for PLAIG’s architecture since it is based on interaction features. Higher-quality protein–ligand complexes in the refined set may have more consistent and well-defined interaction features that correlate strongly with their binding affinities. PLAIG can capture these relationships and make accurate predictions at a higher rate in the refined set than with complexes from the general set.

#### 3.3.3 Model Evaluation Based on the Combined Set

The final two models were trained and tested on the combined set from the PDBbind v.2020 data set. The combined set is a combination of the refined and general sets, serving as the base training data set for the standalone version of PLAIG due to its size and diversity. Users can test this version of PLAIG at: https://plaig-demo.streamlit.app/. The number of topological protein and ligand features input into these models were the same as the number of features in the refined and general versions. To keep the runtime of this model manageable for multiple simulations, we decided to limit the number of complexes in the combined set to the first 10,000 randomly chosen from both the general and refined sets. After filtering out complexes that threw errors, the data set contained approximately 7600 complexes for training and testing. Figure 7 contains the performance metric boxplots for the pre-PCA and post-PCA models trained on the combined set with 10-fold cross validation, including the boxplots of the runtime per fold in minutes.

**Figure 7:**
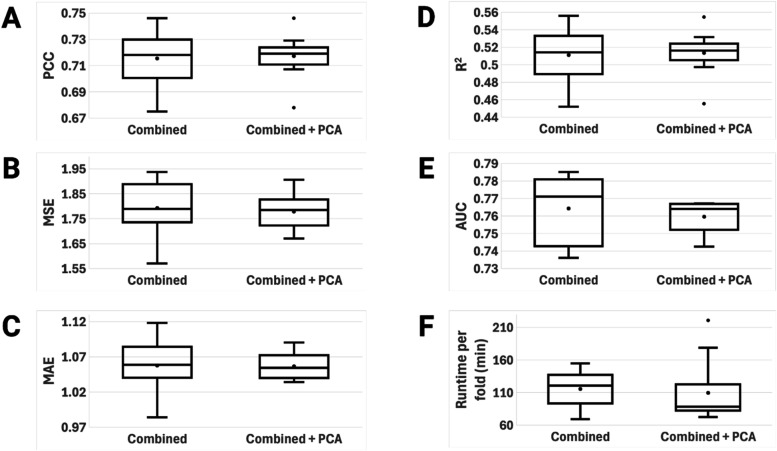
PLAIG’s performance metrics for the combined set comparing the model before and after principal component analysis was implemented. (A) PCC, (B) MSE, (C) MAE, (D) *R* ^2^, (E) AUC, (F) Runtime per fold (min).

The boxplots show average PCCs of 0.715 and 0.717, average MSEs of 1.792 and 1.778, average MAEs of 1.058 and 1.056, average *R* ^2^s of 0.511 and 0.513, and average AUCs of 0.764 and 0.760 for the pre-PCA and post-PCA models, respectively. We see no significant difference in performance metrics after applying PCA, which is the same trend observed with models trained on the other subsets of data. The combined set models rank in the middle when compared to the refined and general models. This provides some evidence that the training and testing performance of PLAIG depends more heavily on the resolution of the .pdb files rather than the size and diversity of the data set. The combined set is bigger than both the refined and general sets, thus containing more noisy data, but it also contains a higher ratio of high-quality complexes than the general set. As a result, this data set contains more accurate biochemical data, which increases the predicted binding affinity accuracy. This is the reason why we chose the combined set to be the main data set that PLAIG is trained on. It has the balance of data diversity/noise and high resolution protein–ligand complexes needed for a robust deep learning model.

### 3.4 External Validation on the Refined and General Sets

After training and testing PLAIG on various subsets of data, we moved on to the external validation phase. We conducted external validation for two main reasons. First, to determine if PLAIG can generalize well to unseen data, and second, to evaluate whether or not the models that incorporate PCA are more robust compared to those without PCA. We tested two out of the three models trained on the PDBbind v.2020 subsets against each other, the refined set and the general. Specifically, we trained one model on the refined set and obtained metrics after testing on the general set, and vice versa. Table 4 shows the metrics for the external validation stage of PLAIG.

**Table 4:** Performance Metrics from External Validation of PLAIG Using the Refined and General Sets^a^

model	PCC	MSE	MAE	*R* ^2^	AUC
PLAIG_ref_	0.577	2.536	1.244	0.331	0.701
PLAIG_refPCA_	0.046	5.212	1.863	–0.376	0.473
PLAIG_gen_	0.671	2.105	1.148	0.446	0.749
PLAIG_genPCA_	0.234	4.108	1.642	–0.080	0.541

a PLAIG_ref_ and PLAIG_refPCA_ are the two models that were trained on the refined set and tested on the general set, while PLAIG_gen_ and PLAIG_genPCA_ were trained on the general set and tested on the refined set.

Looking at the table, we can draw two conclusions. One is about the robustness of the models with PCA. The PCCs for PLAIG_refPCA_ and PLAIG_genPCA_ are much lower than expected, at 0.046 and 0.234, respectively. Additionally, the negative *R* ^2^ values of −0.376 and −0.080 indicate that these models perform worse than simply predicting the mean binding affinity for every complex. This shows that the models post-PCA cannot accurately predict the binding affinity of unseen data. The reason for this could be due to two possible scenarios. PCA focuses on preserving the explained variance of the data; however, this does not always conserve predictive power. It is possible that when reducing dimensionality, PCA removed the features that were highly correlated with the binding affinity predictions, thus losing predictive power. Another explanation for the lack of predictive power is that the features used in PLAIG are not linearly related. PCA assumes a linear relationship between features, but when there are nonlinear relationships, it cannot capture these relationships effectively.^31^

The second conclusion that we can draw from the table is that PLAIG, without PCA, can generalize well to unseen complexes. The PCCs of PLAIG_ref_ and PLAIG_gen_ are 0.577 and 0.671, respectively. Although there is about a 0.1–0.2 drop in PCC, this is expected for any type of external validation. In particular, we can see that the drop in PCC is more significant for PLAIG_ref_, which is trained on the refined set and tested on the general. This makes sense when we see that the general set is a much more diverse and large data set than the refined. In training, PLAIG_ref_ was not exposed to the range of binding affinities that are present in the general set. During the testing phase, it was unable to predict high and low binding affinities because it is not familiar with predictions of extreme magnitudes. PLAIG_gen_ had a much better performance with only about a 0.05 dropoff in PCC. Since this model was trained on the diverse general set, it was more robust and could accurately predict the binding affinity of unknown complexes in the refined set. After analyzing this initial external validation stage with PLAIG, we decided that we would not be moving forward with further validation of the models with PCA. However, we did decide that the standalone PLAIG trained on the combined set (general and refined complexes) could be successful in additional external validation phases and ultimately with future de novo testing.

### 3.5 External Validation Using the DUDE-Z Data Set

An additional validation step was taken to confirm the discriminative power of PLAIG using the DUDE-Z data set. The DUDE-Z data set is the next version of the DUD-E database of decoys that contains 43 receptors with their native crystal structure ligand and thousands of active and decoy ligands.^24^ The key aspect of the DUDE-Z data set is that receptor and ligand files are included in docked conformations. This allows PLAIG to predict the binding affinity of the receptor–ligand complexes without an additional docking step in the workflow. After converting all the files to .pdb and .pdbqt format, we created graph representations of each protein–ligand complex and fed them through PLAIG_com_ (trained on the combined set). The model outputs the binding affinity for each complex as −log­(*K*_d_/*K*_i_) values. Data tables with predictions for all the receptor–ligand complexes from DUDE-Z by receptor type are in Table S2. This file shows the protein–ligand pairs from DUDE-Z, as well as their predicted binding affinity in −log­(*K*_d_/*K*_i_) and micromolar (μM). Ligands with “ZINC” in the name are annotated as active ligands, while ligands with other names are marked as active ligands. The ligands marked as “xtal” are the native ligands for the receptor.

Looking at the tables by themselves, it is hard to determine if PLAIG was able to distinguish between active and decoy ligands. To further quantify PLAIG’s discriminative power, we made violin plots and conducted *t* tests for the active and decoy ligand binding affinity predictions. The predicted binding affinities in −log­(*K*_d_/*K*_i_) are indicated in the *y*-axis of the violin plots. In this case, a binding affinity of 6 or 1 μM is the cutoff value between true active and true decoy. Any value above 6 or higher is considered the range for a true active, while below 6 is a true decoy. In addition, we used this cutoff value to calculate ROC curves that depict the AUC of PLAIG. Figure 8 presents an ROC curve and violin plot for analyzing the external validation results of the overall DUDE-Z data set, along with corresponding plots for the individual FA7 receptor. Figures S5 and S6 contain the ROC curves and violin plots for all 43 receptors of the DUDE-Z data set, respectively.

**Figure 8:**
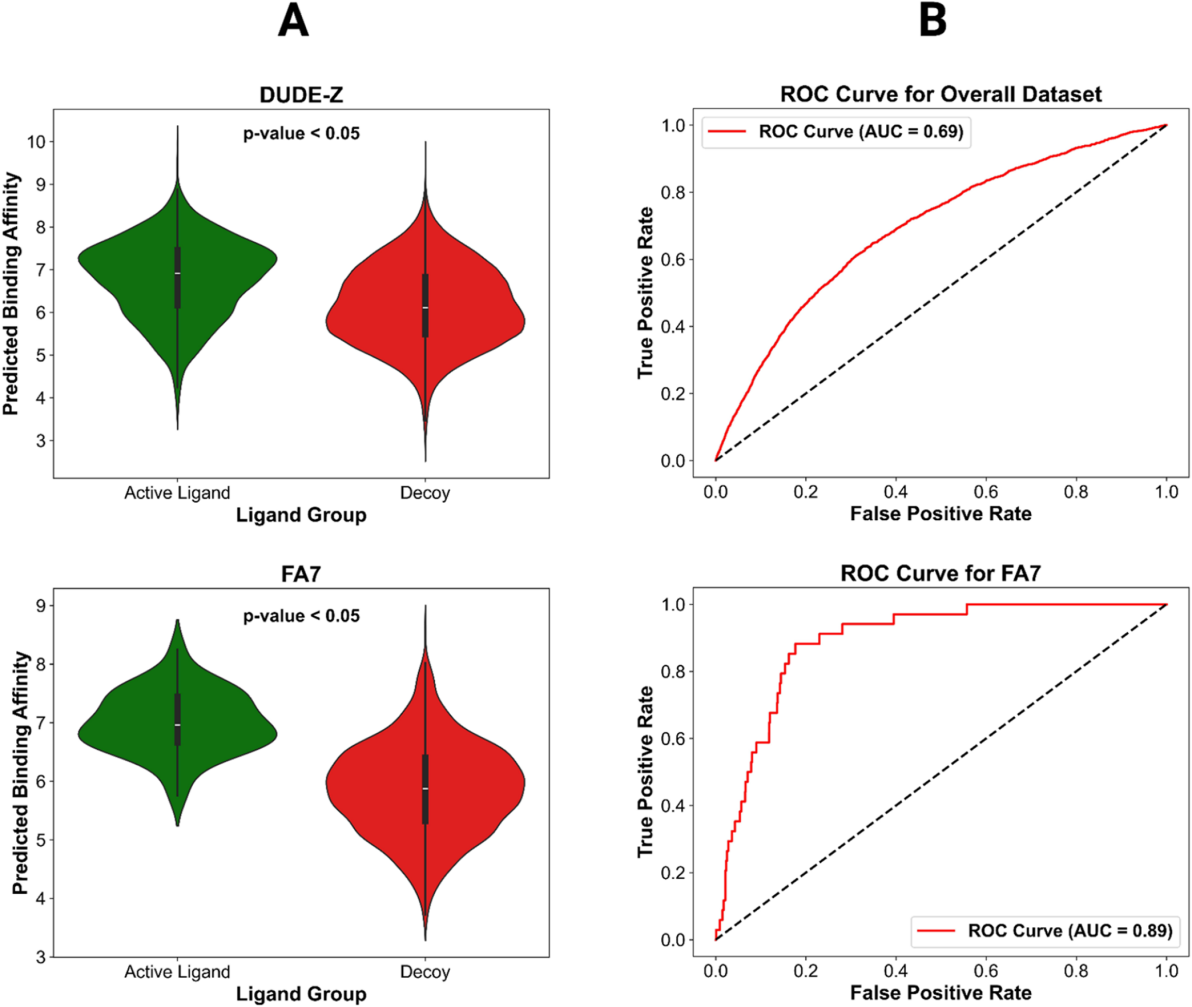
(A) Violin plots that show the distribution of affinity predictions for the active and decoy ligands. Locations on the plot with a wide color density indicate a higher frequency of predictions. The *p*-value in the top center of the plots are from *t* tests comparing the binding affinity prediction means between the active and decoy ligands. The top plot is for the overall data set and the bottom is for active and decoy ligands against the FA7 (coagulation factor VII) protein. (B) ROC curves that show the relationship between the true positive rate (TPR) and the false positive rate (FPR). The 45° angle dotted line going through the center of the graph is the baseline that shows what a perfectly random classifier would predict (TPR = FPR). Just like the violin plots, the top graph is for the overall data set, while the bottom graph is for the FA7 protein.

The violin plots show that on average for the entire DUDE-Z data set, PLAIG predicted active ligands with stronger binding affinities to their receptors than decoy ligands. This difference in binding affinity predictions is also statistically significant, since the *p*-value for the *t* test is less than 0.05. For quantifying PLAIG’s discriminatory power, while the exact cutoff value between active and decoy binding affinities may vary across receptors in the data set, setting the binding affinity threshold at 1 μM results in an overall AUC of 0.69. For specific receptors, PLAIG’s AUC can reach as high as 0.89. The DUDE-Z data set was designed to test the capability of a model to correctly classify the activation potential of different ligands that have subtle variations in their conformation with the binding pocket. This shows that PLAIG’s unique framework has the power to learn important patterns about the chemical interactions and topological features that describe protein–ligand complexes and use this information to make accurate binding affinity predictions.

### 3.6 Ablation Study on Interaction and Molecular Structure Features

To evaluate the impact of our uniquely stored feature categories, we conducted an ablation study by systematically removing specific sets of features from PLAIG and assessing the resulting changes in performance. We trained and tested four variants of PLAIG on the refined set using 10-fold cross-validation: the full model with all features, a model without BINANA-derived interaction features, a model without global topological structural features, and a model with only basic atomic node-level features. The results of this study are shown in Table 5.

**Table 5:** Metrics from Ablation Study on Interaction and Molecular Structure Features^a^

model variant	PCC	MSE	MAE	*R* ^2^	AUC
PLAIG	0.763	1.564	0.997	0.581	0.774
PLAIG_NI_	0.611	2.444	1.223	0.348	0.725
PLAIG_NS_	0.664	2.095	1.155	0.439	0.746
PLAIG_NIS_	0.460	2.848	1.306	0.140	0.653

a PLAIG refers to the full model incorporating all features. PLAIG_NI_ is a variant of PLAIG that excludes interaction features, while PLAIG_NS_ leaves out global structural features. PLAIG_NIS_ contains neither interaction nor global structural features and relies solely on basic node-level atomic information.

Table 5 indicates that the full model achieved the highest PCC of 0.763. Removing interaction features led to a significant drop in performance, reducing the PCC to 0.611, while removing structural features resulted in a slightly less significant drop in PCC to 0.664. The model with only basic atomic features performed the worst, with a PCC of 0.460. These findings highlight the importance of both our interaction and global structural features in PLAIG’s predictive accuracy. However, the greater decline in PCC when interaction features were removed suggests that chemical interactions play a more critical role in the model’s overall performance than topological features.

### 3.7 Benchmarking of PLAIG against Interaction-Based Models

Following the external validation of PLAIG, we compared it with other protein–ligand binding affinity prediction tools that use interaction features in their calculations.^6,9–19^ The metrics reported in previous studies reflect each model’s performance on data sets that were not used during training, providing a more detailed assessment of the model’s generalization and robustness. We used three data sets to benchmark our model against other studies: the PDBbind v.2019 refined set, the PDBbind v.2016 core set, and the PDBbind v.2013 core set. The PDBbind core sets are smaller, high-quality data sets, each containing approximately 100–200 protein–ligand complexes. Table 6 presents the performance metrics for each deep learning model from our study and other relevant literature, along with the data sets tested.

**Table 6:** Benchmarking of PLAIG’s Performance Metrics against Interaction-Based Models^a^

model	protein–ligand test data	PCC	MSE	MAE
PLAIG_com2_	PDBbind v.2019 refined set	0.784	1.484	0.945
PLAIG_com2_	PDBbind v.2016 core set	0.816	1.685	1.005
PLAIG_com2_	PDBbind v.2013 core set	0.710	2.708	1.321
HNN-denovo	PDBbind v.2019 refined set	0.840	0.922	N/A
TopBP	PDBbind v.2016 core set	0.861	N/A	N/A
Hac-Net	PDBbind v.2016 core set	0.846	N/A	0.971
Pafnucy	PDBbind v.2016 core set	0.780	2.016	1.130
Pafnucy	PDBbind v.2013 core set	0.700	2.624	N/A
DeepBindRG	PDBbind v.2013 core set	0.639	3.302	1.483
DeepAtom	PDBbind v.2016 core set	0.831	1.518	0.904
SIGN	PDBbind v.2016 core set	0.797	1.836	1.027
LigityScore	PDBbind v.2016 core set	0.725	2.277	1.224
LigityScore	PDBbind v.2013 core set	0.713	2.809	1.335
OnionNet	PDBbind v.2016 core set	0.816	1.633	0.984
OnionNet	PDBbind v.2013 core set	0.782	2.259	1.208
DLSSAffinity	PDBbind v.2016 core set	0.790	1.960	N/A
PIGNet	PDBbind v.2016 core set	0.761	N/A	N/A
GIGN	PDBBind v.2013 core set	0.821	1.904	N/A

a PLAIG_com2_ refers to the version of PLAIG trained on the combined data set, excluding complexes with PDB IDs in the PDBbind v.2013 and v.2016 core sets.

The most commonly reported metrics were the PCC, MSE, and MAE. PLAIG_com_ achieved PCC values of 0.784 on the PDBbind v.2019 refined set, 0.816 on the PDBbind v.2016 core set, and 0.710 on the PDBbind v.2013 core set. The corresponding MSE values were 1.484, 1.685, and 2.708, while the MAE values were 0.945, 1.005, and 1.321, respectively. In addition, PLAIG averaged a runtime of 1.52 s when predicting the affinity of one protein–ligand complex. For the PDBbind v.2016 core set, PLAIG performed on par with or better than most existing models, except for DeepAtom, TopBP, and Hac-Net, which achieved PCCs of 0.831, 0.861, and 0.846, respectively.^9,10,13^ Notably, TopBP did not use a validation set during testing, which may have led to an inflated PCC. On the PDBbind v.2013 core set, PLAIG also demonstrated strong performance, outperforming or almost matching all models except GIGN.^19^ On the PDBbind v.2019 refined set, PLAIG did not perform as well as the HNN-denovo model. However, it is important to note that the HNN-denovo model was trained specifically on the PDBbind v.2019 refined set and evaluated on a subset of that data set which was excluded before training and validation. In addition, the HNN-denovo model is a merged model which uses a combination of protein sequences, ligand SMILES, and protein–ligand interaction features.^6^

### 3.8 Evaluation of PLAIG Hybridized with External Models

In line with our main goal of accurate virtual screening and de novo drug affinity prediction using PLAIG’s deep learning framework, we conducted preliminary testing for improvements by hybridizing PLAIG with the external models discussed in Section 2.8. To determine whether the incorporation of these external models improved PLAIG’s predictive capabilities, we retrained and evaluated each hybridized model using a 5-fold cross validation of the refined PDBbind v.2020 data set containing approximately 5000 complexes. This approach allowed for a direct comparison with the standalone version of PLAIG. Additionally, we optimized the algorithmic hybrids with Hyperopt. The optimal model with sequence-based features used a single fully connected layer with 256 hidden channels. The model hybridized with the external structural features replaced PLAIG’s ligand and protein topological structure features with fully connected layers of size 256. Table 7 shows the performance metrics for each hybridized model we created.

**Table 7:** Metrics from the Hybridization with External Models Using 5-Fold Cross Validation^a^

merged model	PCC	AUC	runtime per fold (min)
PLAIG_ref_	0.759	0.775	98.2
PLAIG_ref_ + Seq_alg_	0.762	0.782	168.4
PLAIG_ref_ + Seq_st_pred_	0.747	0.774	104.3
PLAIG_ref_ + Seq_st_embed_	0.763	0.779	158.6
PLAIG_ref_ + Str_alg_	0.757	0.777	54.3
PLAIG_ref_ + Seq_st_embed_ + Str_alg_	0.762	0.775	46.4

a PLAIG_ref_ is the standalone version of PLAIG trained on the refined data set. Seq_alg_ is the external sequence model algorithmically hybridized into PLAIG’s GNN. Seq_st_pred_ and Seq_st_embed_ indicate that the sequence model is stacked with either PLAIG’s affinity predictions or GNN embeddings, respectively. Similarly to Seq_alg_, Str_alg_ is the model with external structural features hybridized into PLAIG’s GNN.

From the data table, we see that the model hybridized with the sequence-based model, PLAIG_ref_ + Seq_st_embed_, slightly outperforms PLAIG_ref_ + Seq_alg_, with PCCs of 0.763 and 0.762, respectively. While the difference in PCC is minimal, the strength of the stacking model lies in its runtime. The stacking version is more efficient, with nearly 1 h less in training and testing time, allowing for quicker predictions. PLAIG_ref_ + Seq_st_embed_ outperforms PLAIG_ref_, as the addition of protein sequence and ligand SMILES features provides more information for distinguishing protein–ligand complexes, which enhance affinity predictions.

Since we believed that stacking with the pure structural features would reduce the accuracy of the overall model, we replaced our topological structural features with the features gathered from the external structural model to create the PLAIG_ref_ + Str_alg_ model. This model achieved a PCC of 0.757, slightly lower than the standalone version of PLAIG with a PCC of 0.759. However, the runtime of PLAIG_ref_ + Str_alg_ is significantly faster than PLAIG_ref_, with runtimes per fold of 54.3 and 98.2 min, respectively. The introduction of the external structural features drastically improved the efficiency of the model and kept the high performance of the model. The external structural features were more complete and covered a broader range of topological and physicochemical properties. The use of sparse matrices likely also contributed to improved efficiency by reducing computational load associated with zero entries.^32^

Finally, the last hybridized model that we tested was PLAIG_ref_ + Seq_st_embed_ + Str_alg_. This model hybridized PLAIG with sequence and structural features, effectively maximizing an extensive range of biochemical information that describes the protein–ligand complex. The average PCC and runtime per fold of this model is 0.762 and 46.4 min. The PCC is essentially the same as PLAIG_ref_ + Seq_st_embed_; however, similar to PLAIG_ref_ + Str_alg_, the runtime is significantly faster. Based on these results, we can assume that the final merged model takes the benefits from both the sequence and structural models. We can see this in the increased accuracy and higher efficiency.

After the training and testing phase, we performed a small external validation phase with the best hybridized models on a small subset of well-known drugs: mebendazole, sulindac, and sunitinib. The 3D-coordinates for these drugs docked with their receptors was downloaded from the RCSB Web site, and their experimental binding affinities were obtained from the BindingDB database. Before predicting the binding affinity of these drugs to their targets, we trained 3 distinct models for prediction: PLAIG_com_, PLAIG_com_ + Seq_st_embed_, PLAIG_com_ + Str_alg_, and PLAIG_com_ + Seq_st_embed_ + Str_alg_. These four models were considered the best based on their robustness. Figure 9 presents the results from this small external validation phase on 12 drug–target complexes. Table 8 has the PCCs for the predictions of each model and Table S3 shows the exact predicted binding affinities for the protein–ligand complexes in a tabular format. PDB codes 2kaw, 3rx3, 3u2c, and 4wev contained the drug sulindac, codes 3g0f, 4agd, 4ks8, 4qmz, 6jok, 6nfz, and 6ng0 contained sunitinib, and the last code, 7odn, was the sole PDB complex to contain mebendazole.

**Figure 9:**
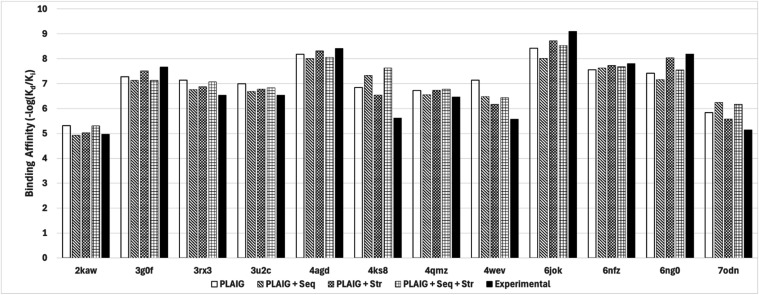
Side-by-side bar chart comparing the binding affinity predictions in −log­(*K*_d_/*K*_i_) of the hybridized models with the experimental values.

**Table 8:** Performance Comparison of Each Hybridized Model during Drug–Target Testing

model	PCC
PLAIG	0.89
PLAIG + Seq	0.82
PLAIG + Str	0.98
PLAIG + Seq + Str	0.85

We can see that while all the models gave predictions close to the experimental value, PLAIG + Str performed the best overall with a PCC of 0.98. The second best model seems to be PLAIG by itself, while the models hybridized with sequence features performed slightly worse. This is surprising because PLAIG stacked with sequence features had the highest PCC during the training/testing phase. One explanation for this is the design of PLAIG’s deep learning framework. This framework is built around chemical interaction and topological structural features. When PLAIG was combined with the external model’s extensive structural features, these additional features enriched PLAIG’s structural learning. This enhanced PLAIG’s ability to generalize to a broader range of protein–ligand complexes by filling in gaps in structural patterns that the standalone model might have missed. In contrast, PLAIG + Seq used a stacking approach rather than an algorithmic integration. This method combined the predictions from each model without modifying the learning processes. As a result, the PLAIG + Seq stacking hybridization might not have improved learning but simply averaged the predictions. This potentially explains the lower accuracy for this subset of complexes.

### 3.9 Evaluation of PLAIG Predictions against AutoDock Vina

A final evaluation was carried out to compare the performance of PLAIG models against AutoDock Vina. Since PLAIG currently requires docked complexes for affinity predictions, we tested how closely PLAIG’s predictions match to experimental data compared to Vina. We ran 12 protein–ligand complexes that were virtually docked by Vina through each of the PLAIG models (PLAIG, PLAIG + Seq, PLAIG + Str, PLAIG + Seq + Str). Vina generates a binding free energy value for each complex after performing virtual protein–ligand docking.^33^ We converted this free energy into binding affinity using the Gibbs Free Energy Change equation

1 $$K_{d}=e^{\Delta G/\mathrm{RT}}$$

where *K*_d_ is the binding affinity in molar, Δ*G* is the free energy prediction from Vina in units of kcal/mol, *R* is the gas constant in units of kcal/(mol·K), and *T* is the temperature in Kelvin at standard state conditions (298 K). Figure 10 shows the predictions from each model represented as fitted linear trendlines.

**Figure 10:**
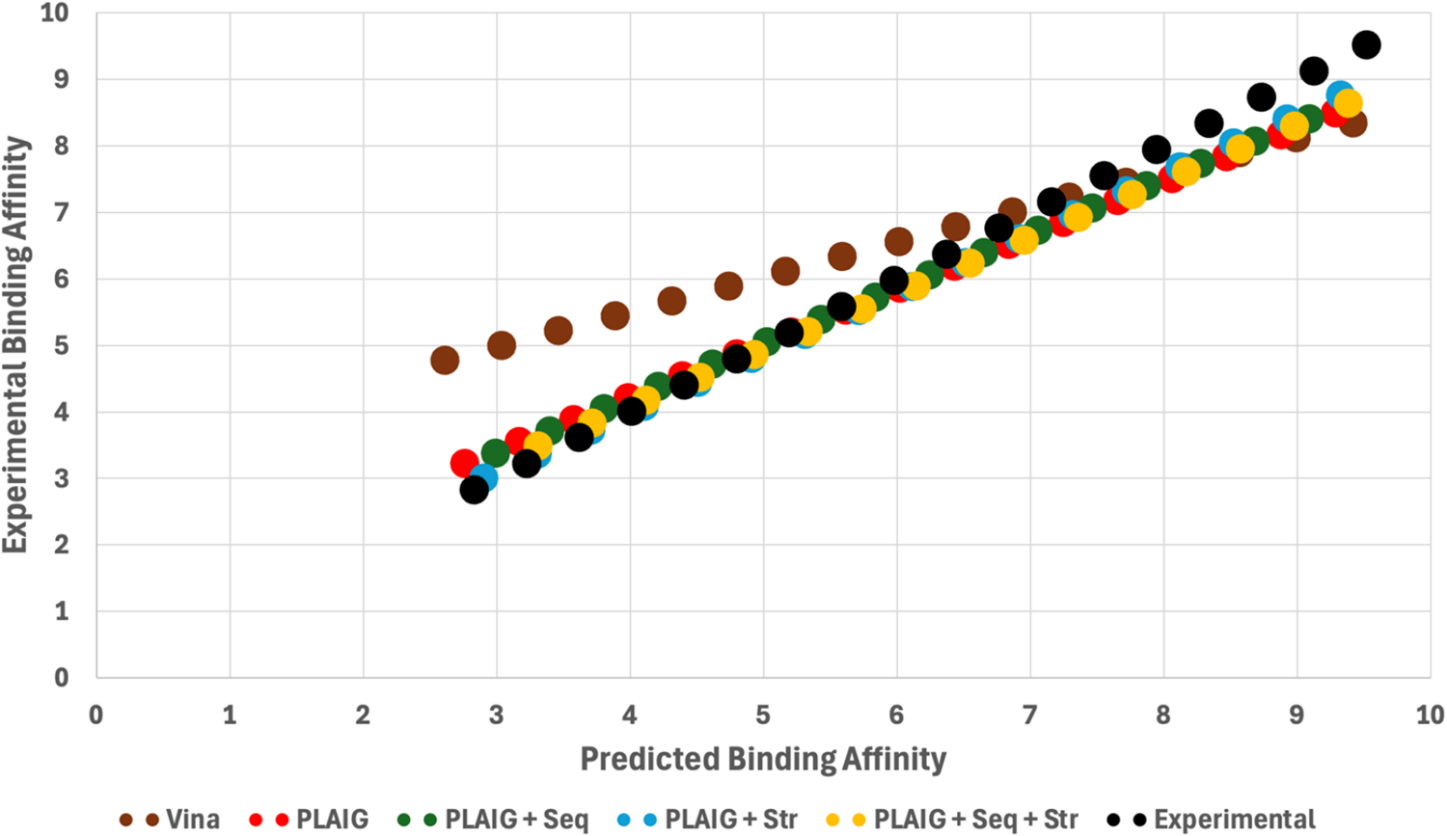
Linear trendlines showing the relationship between experimental and predicted binding affinities for four models (PLAIG, PLAIG + Seq, PLAIG + Str, PLAIG + Seq + Str) and AutoDock Vina. The binding affinity values are represented in -log­(K_d_) for smoother linear fit calculations. Each trendline was calculated based on predictions for 12 complexes docked by Vina. The black dotted line represents the experimental reference line, indicating perfect predictions (no error).

Each trendline is compared against the reference, which represents a fitted line based on the experimental data. We evaluated the performance of each model on the 12 docked complexes by observing how closely each trendline aligns with the reference. From the plot, we can see that the PLAIG models are closer to the reference compared to the Vina trendline. The PCC values were 0.64 for Vina, 0.72 for PLAIG, 0.74 for PLAIG + Seq, 0.85 for PLAIG + Str, and 0.72 for PLAIG + Seq + Str. These trendlines and PCC values provide evidence that PLAIG produces more accurate binding affinity predictions than AutoDock Vina. Although PLAIG cannot predict binding affinities for undocked complexes, it proves useful for achieving higher accuracy when complexes are virtually docked. Binding affinity predictions for the 12 docked complexes by PLAIG’s standalone model and Vina compared to the experimental can be demoed at https://plaig-demo.streamlit.app/.

While these hybridized models performed well during the training/testing and external validation phases, our ultimate goal is to use PLAIG for de novo drug predictions and rapid virtual screening for supplementary analysis. The results from this section and Section 3.8 demonstrate that PLAIG has promise for performing well during de novo testing, even if we cannot perform this kind of testing at this time.

## 4 Limitations

PLAIG performed well at predicting protein–ligand binding affinities but faces limitations due to its architecture and input requirements. Its GNN framework involves multiple layers and separate processing of atomic, interaction, and topological features. While this layered design captures complex spatial patterns effectively, it demands high computational resources. This results in training times exceeding an hour per fold with a batch size of 32 and 256 channels per layer. Because of this, scalability is limited when handling larger, diverse data sets.

Another critical limitation is that PLAIG requires predocked protein–ligand complexes in both .pdb and .pdbqt formats to compute interaction features. This poses a challenge for users unfamiliar with format conversions. To streamline this process, we plan to add docking functionality to PLAIG. This way, users can calculate the predicted binding affinity of novel protein–ligand complexes without any experimental 3D crystal structures.

PLAIG predominantly focuses on interaction features and limited structural descriptors, omitting sequence-based information for proteins and ligands. Sequence features, which provide insights into the amino acid composition of the protein, are vital for understanding the binding pocket environment. By neglecting these sequence features, the model potentially misses out on valuable information that could improve its predictive performance.^34^ Finally, PLAIG uses absolute 3D coordinates as node features rather than relative interatomic information. This approach limits the model’s ability to handle translations and rotations of the protein–ligand complex, preventing SE(3) equivariance. Future iterations of PLAIG will explore the use of relative information in place of rigid coordinates.

## 5 Future Directions

PLAIG’s current GNN framework is designed to capture the intricate patterns between protein–ligand complexes that influence binding affinity. We plan to improve this framework by integrating a Graph Attention Network (GAT) model into our base GNN. The self-attention mechanism of GATs can streamline the process of capturing complex patterns to improve both efficiency and performance.^35^ Additionally, incorporating Graph Isomorphism Networks (GINs) will help differentiate between protein–ligand structures by using the Weisfeiler–Lehman (WL) graph isomorphism test. Protein–ligand complexes that are isomorphic likely have similar structures, leading to similar binding affinities.^36^ These changes aim to reduce training times and improve generalization for novel and diverse data sets.

As discussed before, PLAIG uses a limited number of structural features and neglects sequence features. We initiated the addition of these features to PLAIG’s framework toward the end of this study, and we can see that sequence and structural features increase both the efficiency and/or accuracy of PLAIG. However, these models need further research which will be conducted in future studies focusing on de novo predictions. We also plan to integrate docking software, such as GNINA, into PLAIG’s workflow to predict 3D structures of protein–ligand complexes from separate protein and ligand files.^37^ GNINA’s docking score will be added as a feature to improve binding affinity predictions and streamline user workflows. These improvements position PLAIG for accurate de novo predictions, enabling large-scale drug discovery and toxicological studies across diverse chemical spaces.

## 6 Conclusions

In this study, we developed and validated PLAIG, a novel machine learning model combining a GNN with a stacking regressor to predict protein–ligand binding affinities. PLAIG represents chemical interactions and topological structural features as graph-structured data. We trained and tested PLAIG on three subsets of the PDBbind v.2020 data set (refined, general, and combined). We performed PCA on the features extracted from these protein–ligand graphs as additional models for testing. In total, we tested 6 different models for the standalone version of PLAIG and determined the combined set without PCA as the best model. PLAIG outperformed established models, achieving an average PCC of 0.784 on the PDBbind v.2019 refined set and 0.816 on the PDBbind v.2016 core set. Validation on the DUDE-Z data set demonstrated robust discriminative power, with an average AUC of 0.69 across 43 receptors. These results demonstrate PLAIG’s ability to generalize and capture key protein–ligand interactions.

To address limitations, we hybridized PLAIG with models incorporating structure- and sequence-based features. These hybrid models showed improved accuracy on a small data set of well-known drugs with an average PCC of 0.88 and outperformed AutoDock Vina when predicting the affinity of virtually docked complexes. We plan to integrate Graph Attention Networks (GAT) or Graph Isomorphism Networks (GIN) to capture intricate data patterns and incorporate docking software like GNINA to allow de novo predictions. The final model could drastically reduce the time required for large-scale virtual screening of drugs and serve as a valuable tool for the analysis of toxicological agents. Future applications include characterizing the toxic effects of PFAS ligands on human receptors. Given PFAS ligands’ carcinogenic potential, PLAIG could significantly contribute to research on disease mechanisms and potential therapeutic targets.^38^

## Supplementary Material



## Data Availability

PLAIG was developed using Python and can be freely tested through this link: https://plaig-demo.streamlit.app/. If the webpage is asleep, click the wake up button to reload the page for use. This GitHub repository, https://github.com/sivaGU/PLAIG, includes the code for PLAIG’s deep learning model, the demo webpage, and zipped files containing the PDBbind v.2020 refined and general training data sets. The Supporting Information below contains additional data supporting results of this study.

## Notes

Features stored in the graph representation of a protein–ligand complex (Table S1); binding affinity predictions of drug–target complexes using hybridized models (Table S3); example of a protein pocket substructure created from an entire protein file docked with its ligand (Figure S1); visualization of the protein–ligand complex graph representations used for prediction (Figure S2); PCA plots to determine optimal number of features for the general set (Figure S3); PCA plots to determine optimal number of features for the combined set (Figure S4); binding affinity predictions for the DUDE-Z data set (Table S2); all receiver operating characteristic (ROC) curves from the DUDE-Z data set binding affinity predictions (Figure S5), and all violin plots from the DUDE-Z data set binding affinity predictions (Figure S6) (ZIP)
