# Supplementary material for: PLAIG: Protein–Ligand Binding Affinity Prediction Using a Novel Interaction-Based Graph Neural Network Framework
Source: ACS Bio Med Chem Au. 2025 Apr 29;5(3):447–63. doi: 10.1021/acsbiomedchemau.5c00053 (PMC12183606; doi:10.1021/acsbiomedchemau.5c00053)
Supplement: Supplementary file 1 [file bg5c00053_si_002.zip › Supporting Information/Supporting Information.docx]

**Supporting Information for PLAIG: Protein-Ligand Binding Affinity Prediction using a Novel Interaction-Based Graph Neural Network Framework.**

Madhav V. Samudrala^1^, Somanath Dandibhotla^2^, Arjun Kaneriya^3^, Sivanesan Dakshanamurthy^4^*

1. *College of Arts and Sciences, The University of Virginia, Charlottesville, VA, 22903, USA*
2. *College of Engineering and Computing, George Mason University, Fairfax, VA 22030, USA*
3. *College of William and Mary, William and Mary, Williamsburg, VA 23185, USA*
4. *Department of Oncology, Lombardi Comprehensive Cancer Center, Georgetown University Medical Center, Washington, DC 20007, USA*

*Email: [sd233@georgetown.edu](mailto:sd233@georgetown.edu)

**Table of Contents**

**List of Tables**

**Table S1.** Features Included in a Graph Representation of a Protein-Ligand Complex.

**Table S2.** DUDE-Z Dataset Binding Affinity Predictions.

**Table S3.** Binding Affinity Predictions of Drugs from Hybridized Models.

**List of Figures**

**Figure S1.** Example of a protein pocket substructure created from an entire protein file docked with its ligand.

**Figure S2.** Visualization of the protein-ligand complex graph representations used for prediction.

**Figure S3.** Cumulative explained variance plots for ligand and protein features generated from the PDBbind v.2020 general set.

**Figure S4.** Cumulative explained variance plots for ligand and protein features generated from the PDBbind v.2020 general set.

**Figure S5.** Receiver operating characteristic (ROC) curves from the DUDE-Z dataset binding affinity predictions.

**Figure S6.** Violin plots from the DUDE-Z dataset binding affinity predictions.

**Table S1.** Features Included in a Graph Representation of a Protein-Ligand Complex

| **Node Features** | **Edge Features** | **Ligand Features** | **Pocket Features** |
| --- | --- | --- | --- |
| Atomic Number | Electrostatic Energy | LogP | LogP |
| Residue or Ligand Code | Halogen Bond | TPSA | TPSA |
| Hybridization Number | Hydrogen Bond | Asphericity | Asphericity |
| Degree | Hydrophobic Contact | Chi0n | Chi0n |
| Aromaticity | Metal Contact | Chi0v | Chi0v |
| Hydrogens | π-π Stacking | Chi1n | Chi1n |
| Atomic Mass | T-stacking | Chi1v | Chi1v |
| Formal Charge | Salt Bridge | Chi2n | Chi2n |
| Gasteiger Charge | Cation-π | Chi2v | Chi2v |
| X Coordinate | Bond Type | Chi3n | Chi3n |
| Y Coordinate | Distance | Chi3v | Chi3v |
| Z Coordinate |  | Chi4n | Chi4n |
|  |  | Chi4v | Chi4v |
|  |  | Eccentricity | Eccentricity |
|  |  | Hall-Kier Alpha | Hall-Kier Alpha |
|  |  | Inertial Shape Factor | Inertial Shape Factor |
|  |  | Kappa 1 | Kappa 1 |
|  |  | Kappa 2 | Kappa 2 |
|  |  | Kappa 3 | Kappa 3 |
|  |  | Labute ASA | Labute ASA |
|  |  | NPR 1 | NPR 1 |
|  |  | NPR 2 | NPR 2 |
|  |  | Rotatable Bonds | Rotatable Bonds |
|  |  | PBF | PBF |
|  |  | PMI 1 | PMI 1 |
|  |  | PMI 2 | PMI 2 |
|  |  | PMI 3 | PMI 3 |
|  |  | Phi | Phi |
|  |  | Radius of Gyration | Radius of Gyration |
|  |  | Aliphatic Carbocycles | MolMR |
|  |  | Aliphatic Heterocycles | QED |
|  |  | Aliphatic Rings | PEOE VSA 1 |
|  |  | Aromatic Carbocycles | PEOE VSA 2 |
|  |  | Aromatic Heterocycles | PEOE VSA 3 |
|  |  | Aromatic Rings | PEOE VSA 4 |
|  |  | NHOH Count | PEOE VSA 5 |
|  |  | NO Count | PEOE VSA 6 |
|  |  | Fraction of CSP3 | PEOE VSA 7 |
|  |  | Saturated Carbocycles | PEOE VSA 8 |
|  |  | Saturated Heterocycles | PEOE VSA 9 |
|  |  | Saturated Rings | PEOE VSA 10 |
|  |  | Rings | PEOE VSA 11 |
|  |  | MolMR | PEOE VSA 12 |
|  |  | QED | PEOE VSA 13 |
|  |  | PEOE VSA 1 | PEOE VSA 14 |
|  |  | PEOE VSA 2 | SMR VSA 1 |
|  |  | PEOE VSA 3 | SMR VSA 2 |
|  |  | PEOE VSA 4 | SMR VSA 3 |
|  |  | PEOE VSA 5 | SMR VSA 4 |
|  |  | PEOE VSA 6 | SMR VSA 5 |
|  |  | PEOE VSA 7 | SMR VSA 6 |
|  |  | PEOE VSA 8 | SMR VSA 7 |
|  |  | PEOE VSA 9 | SMR VSA 8 |
|  |  | PEOE VSA 10 | SLogP VSA 1 |
|  |  | PEOE VSA 11 | SLogP VSA 2 |
|  |  | PEOE VSA 12 | SLogP VSA 3 |
|  |  | PEOE VSA 13 | SLogP VSA 4 |
|  |  | PEOE VSA 14 | SLogP VSA 5 |
|  |  | SMR VSA 1 | SLogP VSA 6 |
|  |  | SMR VSA 2 | SLogP VSA 7 |
|  |  | SMR VSA 3 | SLogP VSA 8 |
|  |  | SMR VSA 4 | SLogP VSA 9 |
|  |  | SMR VSA 5 | SLogP VSA 10 |
|  |  | SMR VSA 6 | SLogP VSA 11 |
|  |  | SMR VSA 7 | SLogP VSA 12 |
|  |  | SMR VSA 8 | VSA EState 1 |
|  |  | SLogP VSA 1 | VSA EState 2 |
|  |  | SLogP VSA 2 | VSA EState 3 |
|  |  | SLogP VSA 3 | VSA EState 4 |
|  |  | SLogP VSA 4 | VSA EState 5 |
|  |  | SLogP VSA 5 | VSA EState 6 |
|  |  | SLogP VSA 6 | VSA EState 7 |
|  |  | SLogP VSA 7 | VSA EState 8 |
|  |  | SLogP VSA 8 | VSA EState 9 |
|  |  | SLogP VSA 9 | VSA EState 10 |
|  |  | SLogP VSA 10 |  |
|  |  | SLogP VSA 11 |  |
|  |  | SLogP VSA 12 |  |
|  |  | VSA EState 1 |  |
|  |  | VSA EState 2 |  |
|  |  | VSA EState 3 |  |
|  |  | VSA EState 4 |  |
|  |  | VSA EState 5 |  |
|  |  | VSA EState 6 |  |
|  |  | VSA EState 7 |  |
|  |  | VSA EState 8 |  |
|  |  | VSA EState 9 |  |
|  |  | VSA EState 10 |  |

**Table S2.** DUDE-Z Dataset Binding Affinity Predictions

This is provided as an Excel file in the Supporting Information folder, TableS2.xlsx

**Table S3.** Binding Affinity Predictions of Drugs from Hybridized Models

| **PDB Code** | **Drug** | **PLAIG** | **PLAIG + Seq** | **PLAIG + Str** | **PLAIG + Seq + Str** | **Experimental** |
| --- | --- | --- | --- | --- | --- | --- |
| 2kaw | sulindac | 5.31 | 4.93 | 5.02 | 5.30 | 4.97 |
| 3g0f | sunitinib | 7.28 | 7.13 | 7.51 | 7.13 | 7.66 |
| 3rx3 | sulindac | 7.14 | 6.76 | 6.87 | 7.07 | 6.53 |
| 3u2c | sulindac | 7.00 | 6.69 | 6.78 | 6.83 | 6.53 |
| 4agd | sunitinib | 8.18 | 8.00 | 8.32 | 8.05 | 8.41 |
| 4ks8 | sunitinib | 6.85 | 7.33 | 6.54 | 7.62 | 5.62 |
| 4qmz | sunitinib | 6.72 | 6.56 | 6.73 | 6.78 | 6.47 |
| 4wev | sulindac | 7.14 | 6.48 | 6.17 | 6.44 | 5.57 |
| 6jok | sunitinib | 8.43 | 8.01 | 8.72 | 8.53 | 9.1 |
| 6nfz | sunitinib | 7.56 | 7.63 | 7.73 | 7.68 | 7.8 |
| 6ng0 | sunitinib | 7.41 | 7.16 | 8.04 | 7.55 | 8.19 |
| 7odn | mebendazole | 5.84 | 6.24 | 5.58 | 6.17 | 5.14 |


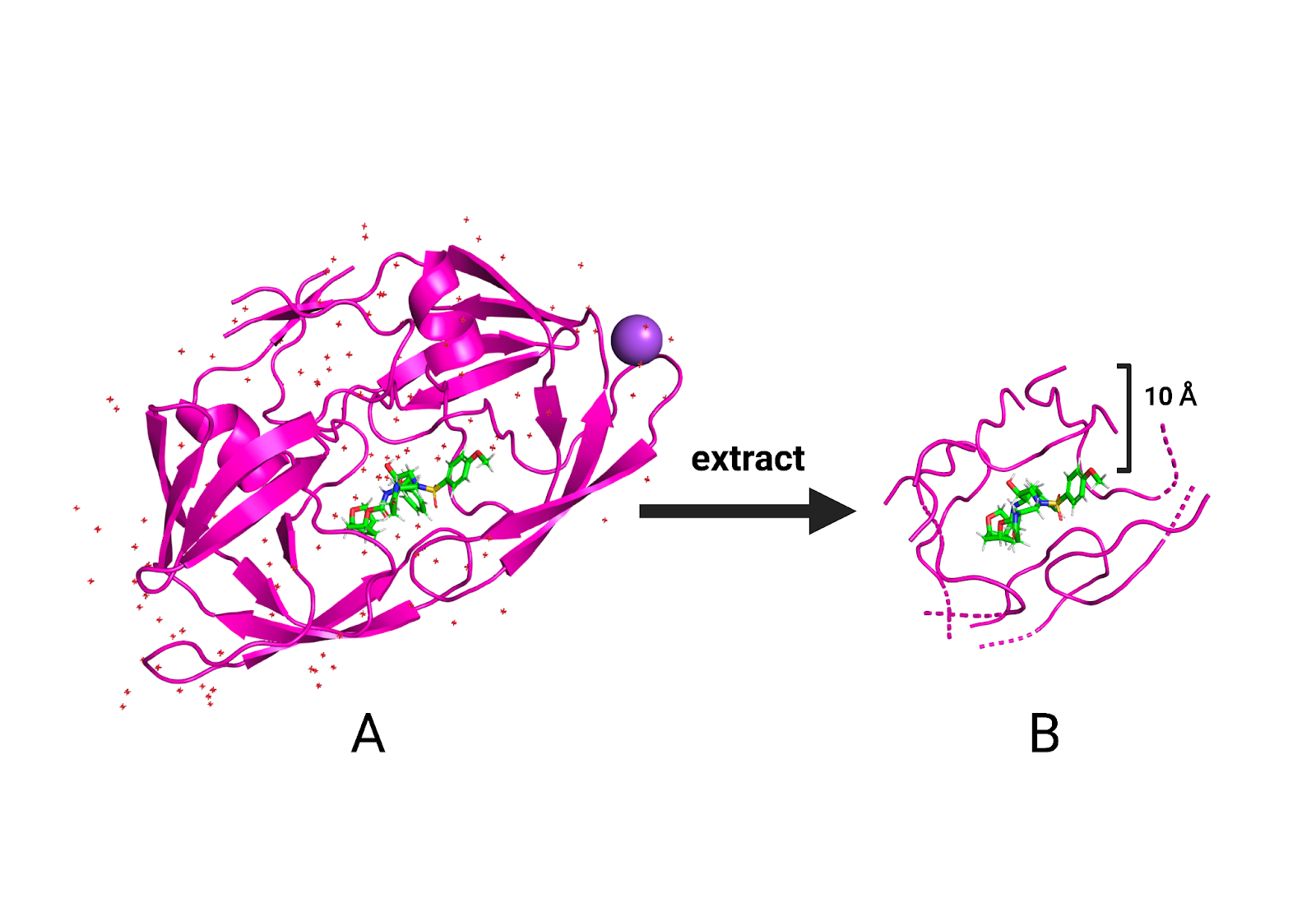


**Figure S1.** Example of a protein pocket substructure created from an entire protein file docked with its ligand. In structure A, the original protein-ligand complex contains all the protein residues in purple, the metal ion in dark purple, and the small water molecules in red. The majority green molecule is the ligand. Structure B is an extracted version of Structure A that includes only the residues within 10 Å of any ligand atom, along with the ligand itself. This is essentially the binding pocket of the original complex. Using the binding pocket, we can focus feature extraction on only the key residues that interact with the ligand. Protein-ligand complex taken from RCSB (PDB code: 3VFA).


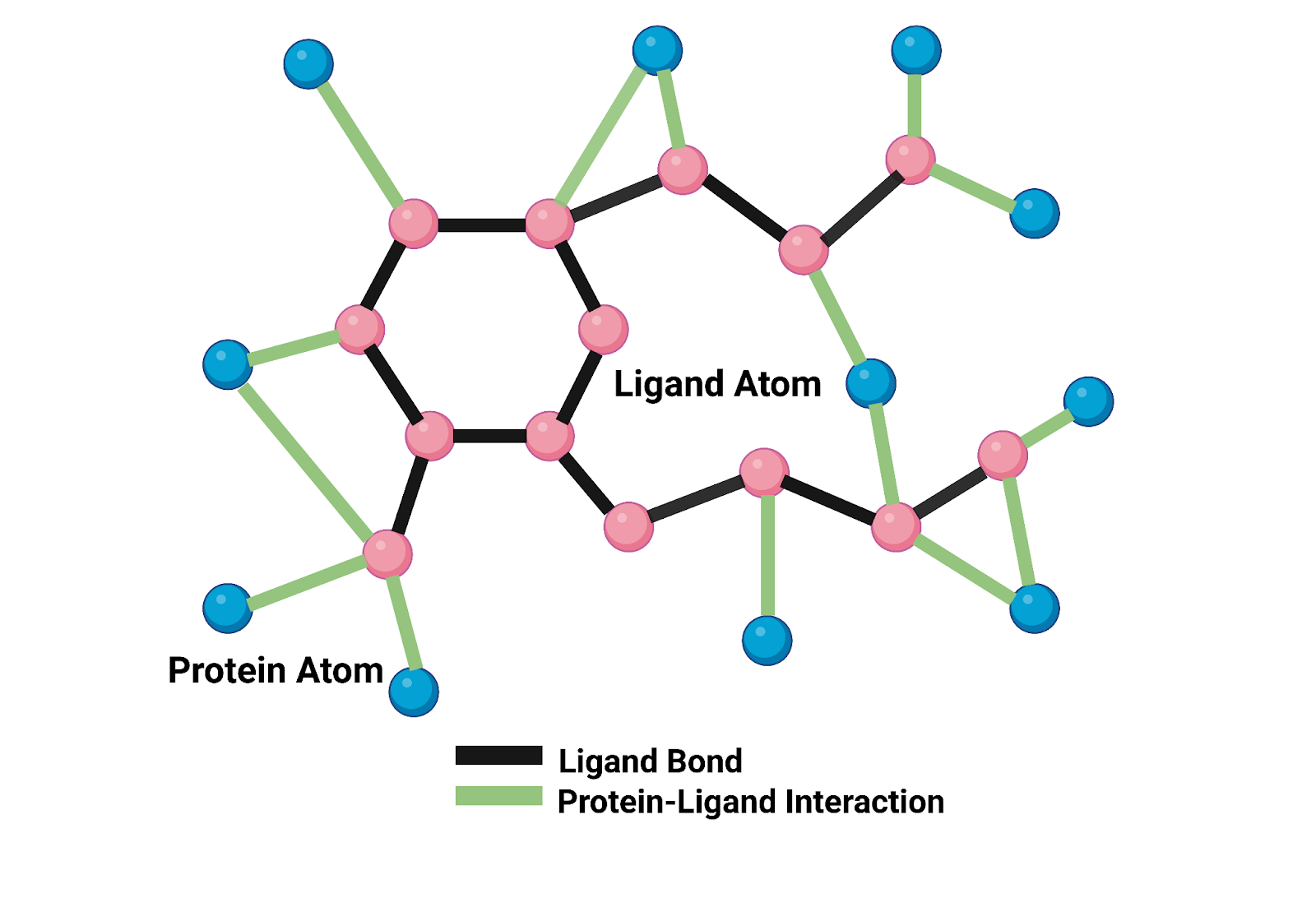


**Figure S2.** Visualization of a graph representing a protein-ligand complex. In the graph, pink nodes correspond to ligand atoms, and blue nodes represent protein atoms that are within 3 Å of any ligand atom. Black edges indicate covalent bonds between ligand atoms, while lime green edges denote potential chemical interactions between protein and ligand atoms. For simplicity, the graph-structured data used in our model does not account for covalent bonds between protein atoms.

**Figure S3.** Cumulative explained variance plots for ligand and protein features generated from the PDBbind v.2020 general set. The figure on the left is the plot for the ligand features, while the figure on the right is for the protein pocket features. The “elbow” point of each graph shows the ideal number of principal components or features needed to capture the necessary information.
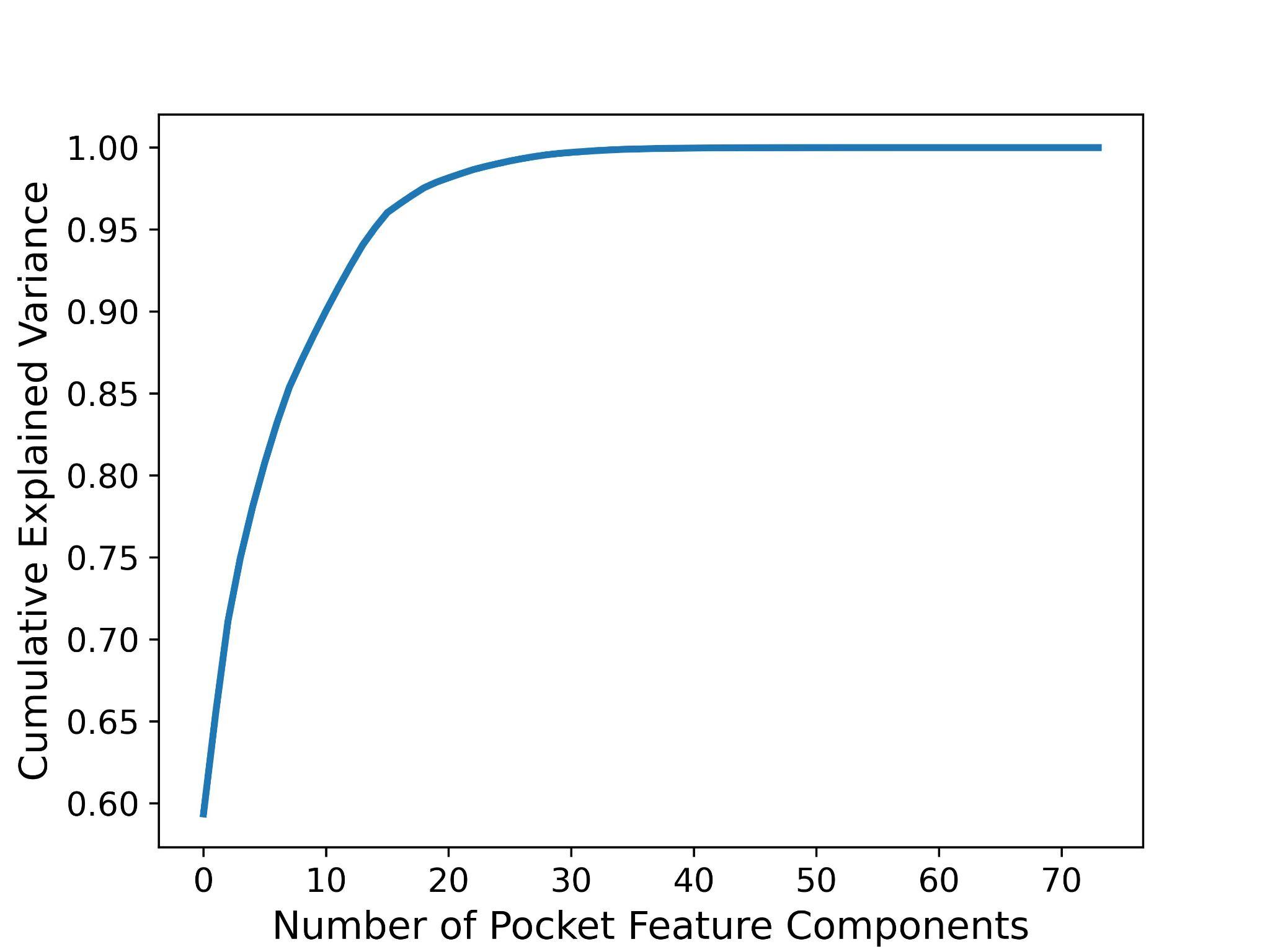

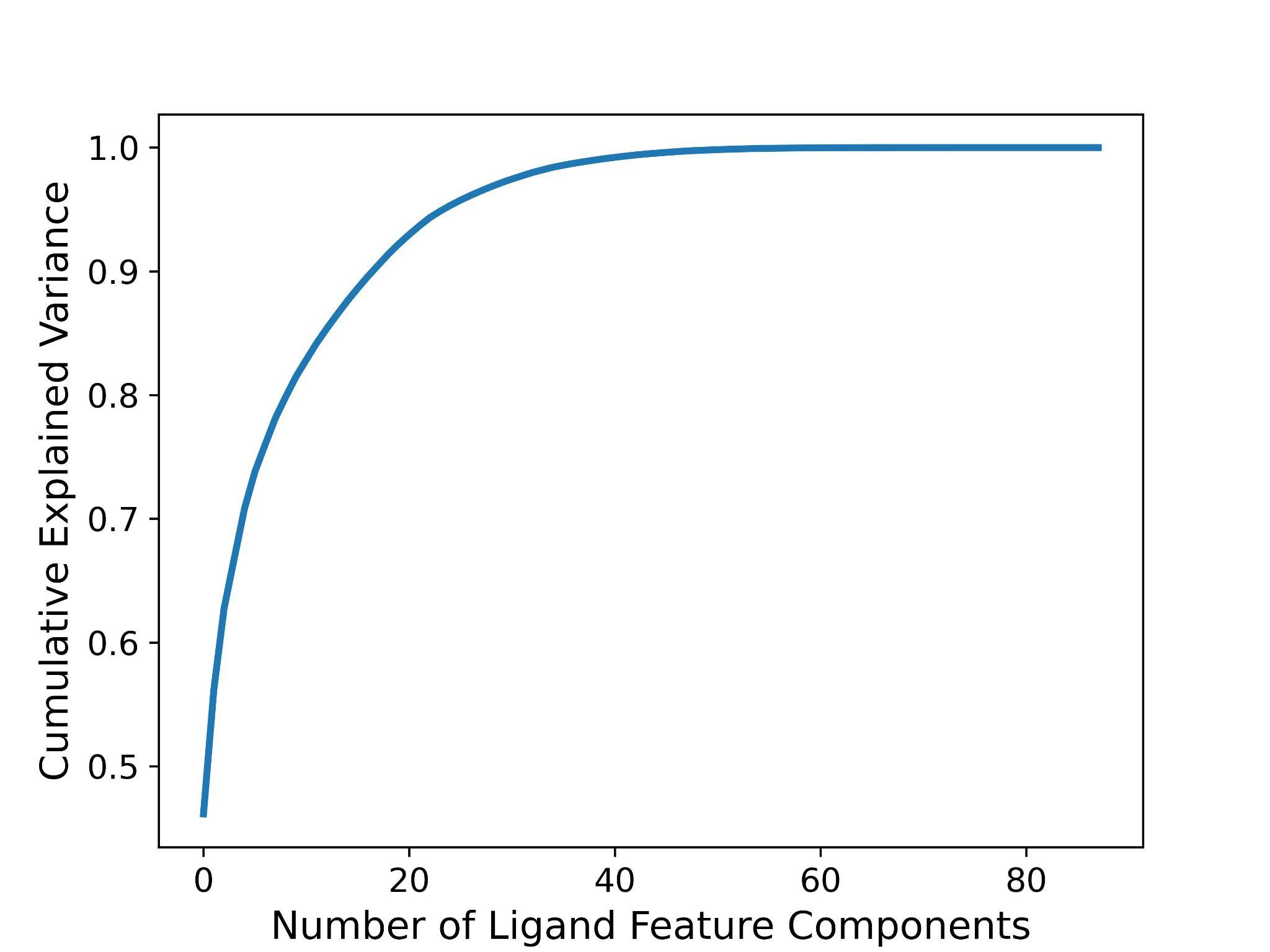


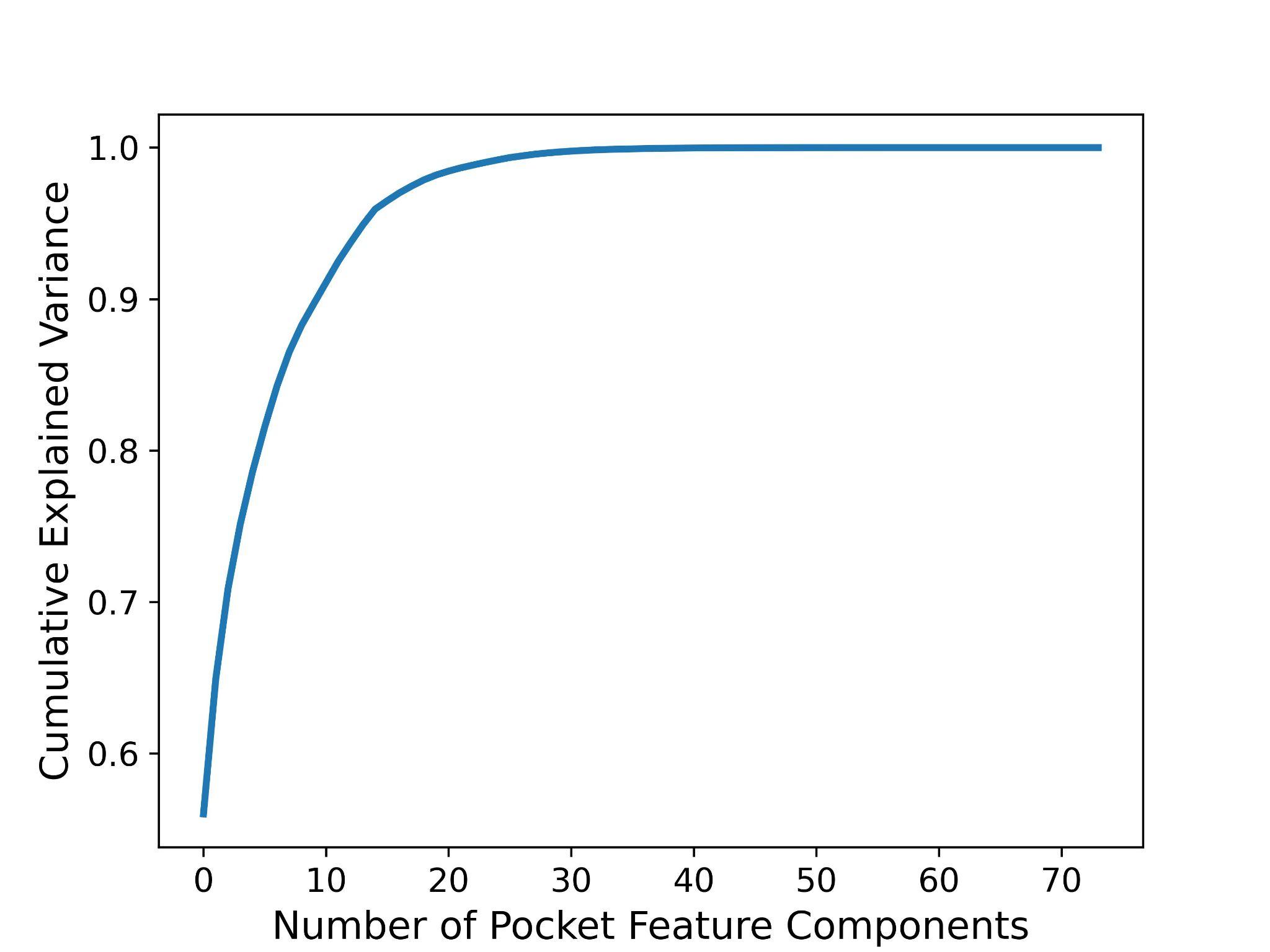

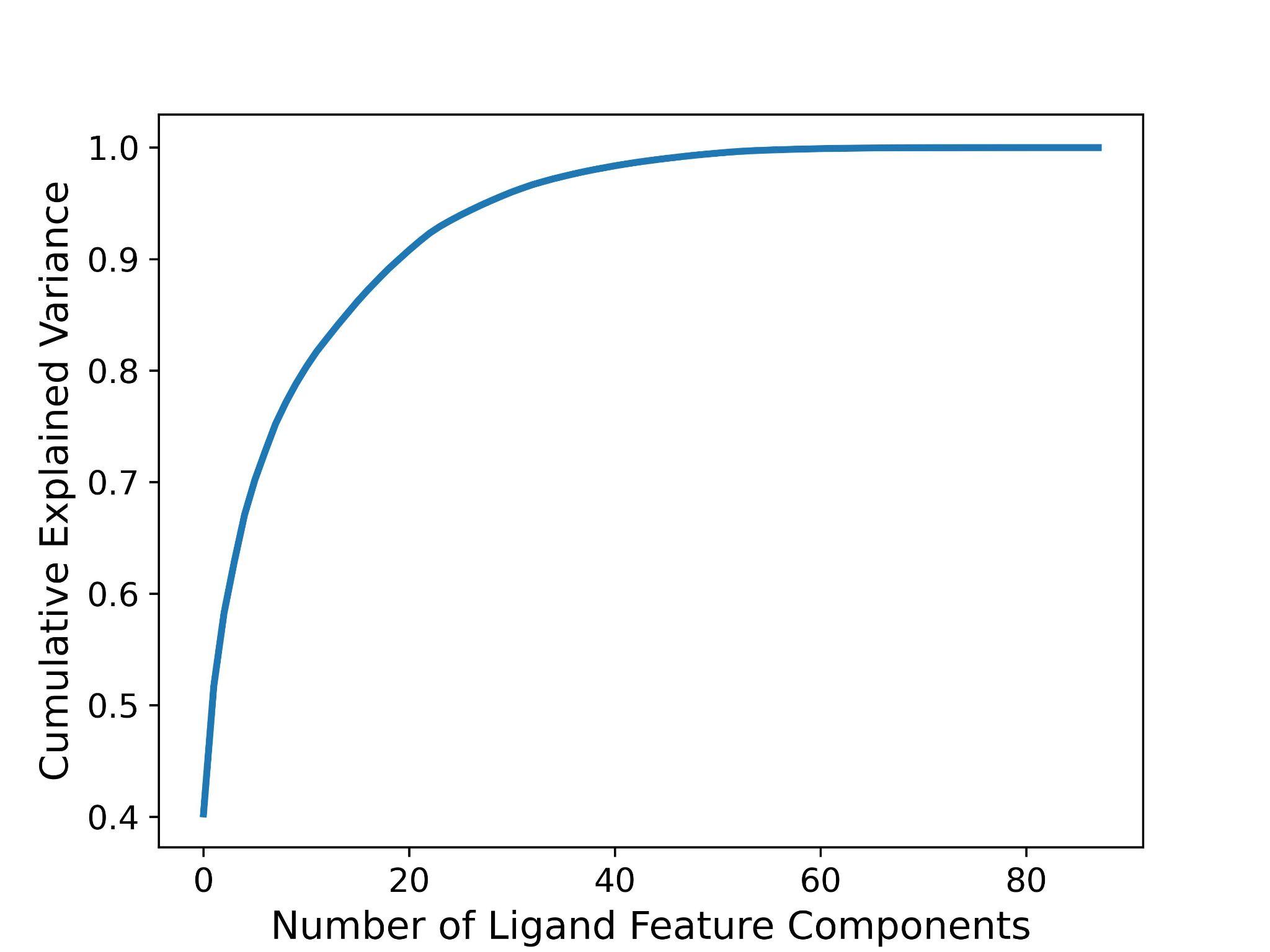


**Figure S4.** Cumulative explained variance plots for ligand and protein features generated from the PDBbind v.2020 combined set. The figure on the left is the plot for the ligand features, while the figure on the right is for the protein pocket features. The “elbow” point of each graph shows the ideal number of principal components or features needed to capture the necessary information.

**Figure S5.** All receiver operating characteristic (ROC) curves from the DUDE-Z dataset binding affinity predictions.

These are located in a zip file in the Supporting Information folder named FigureS3.zip containing multiple figures in JPG format.

**Figure S6.** All violin plots from the DUDE-Z dataset binding affinity predictions.

These are located in a zip file in the Supporting Information folder named FigureS4.zip containing multiple figures in JPG format.
